# Synergistic Self-Healing Enhancement in Multifunctional Silicone Elastomers and Their Application in Smart Materials

**DOI:** 10.3390/polym16040487

**Published:** 2024-02-09

**Authors:** Anna Kowalewska, Kamila Majewska-Smolarek

**Affiliations:** Centre of Molecular and Macromolecular Studies, Polish Academy of Sciences, Sienkiewicza 112, 90-363 Łódź, Poland; kamila.majewska-smolarek@cbmm.lodz.pl

**Keywords:** silicone elastomers, PDMS, self-healing, synergistic enhancement, smart materials

## Abstract

Organosilicon polymers (silicones) are of enduring interest both as an established branch of polymer chemistry and as a segment of commercial products. Their unique properties were exploited in a wide range of everyday applications. However, current silicone trends in chemistry and materials engineering are focused on new smart applications, including stretchable electronics, wearable stress sensors, protective coatings, and soft robotics. Such applications require a fresh approach to methods for increasing the durability and mechanical strength of polysiloxanes, including crosslinked systems. The introduction of self-healing options to silicones has been recognized as a promising alternative in this field, but only carefully designed multifunctional systems operating with several different self-healing mechanisms can truly address the demands placed on such valuable materials. In this review, we summarized the progress of research efforts dedicated to the synthesis and applications of self-healing hybrid materials through multi-component systems that enable the design of functional silicon-based polymers for smart applications.

## 1. Introduction

Siloxane elastomers (silicones) are well-established materials with valued physical and chemical properties (thermostability over a wide temperature range, low surface energy, environmental and electrical resistance, good low-temperature flexibility), as well as biocompatibility [1,2]. The mechanical properties of traditional siloxane elastomers are moderated by adjusting their molecular weight or degree of crosslinking. However, with the rapid advancement of new technologies requiring high-performance siloxane-based polymeric materials, new strategies have emerged to deal with their insufficient mechanical strength by implementing a variety of non-covalent supramolecular interactions and dynamic covalent bonds [3,4,5,6,7]. Silicones with an intrinsic self-healing (S-H) mechanism are promising candidates toward sustainable and recyclable applications. The S-H capability not only extends the service life of these polymers, but also helps meet some specific requirements for smart materials, including stretchable electronics, stimuli-responsive materials, wearable stress sensors, protective coatings, soft robotics and similar new applications [2]. The self-regeneration mechanisms can be chemical or physical in its nature or a combination of both [8,9,10]. Chemical S-H may involve incorporation of dynamic covalent bonds or supramolecular interactions. The physical phenomena behind the S-H processes include most often phase-separated morphologies and inter-chain diffusion, but also the action of superparamagnetic species and shape-memory effects.

Reprocessable S-H silicone elastomers with high mechanical strength are not only very attractive for many applications, but also important with regard to the problem of the reuse of resources. However, simultaneous improvement of mechanical and self-healing properties is difficult to balance and many self-healing polymers require some kind of trade-off between the self-healing effect introduced by strong and weak crosslinking bonds and the mechanical properties of the material [11]. The mechanical strength of silicones can be improved by using macromolecules with shorter chain lengths, or multi-armed species [12]. The functionalization of PDMS chains to produce weaker dynamic bonds and supramolecular interactions does not reduce the motion of the polymer segments, but at the same time makes the material viscoelastic. Silicones crosslinked by non-covalent bonds typically display good flexibility and elasticity; compared to those with covalent crosslinking, their stiffness and mechanical strength can be rather poor. On the other hand, crosslinking through the formation of strong dynamic covalent bonds and metal chelation can, while leading to an increase in the mechanical properties of the polymer material (modulus and tensile strength), also hinder the ability to self-repair due to the acquired mobility limitation of the polymer chains.

For these reasons, the design of self-healing polysiloxanes with an autonomous self-healing capability and exhibiting good mechanical properties still remains a challenge. The significance of this problem can be reduced by using strategies based on the incorporation of multiple dynamic bonds into a single network, the formation of interpenetrating double networks or multiphase systems. The right combination of strong and weak bonds in such complex architectural systems can yield the desired properties. Weak “sacrificial” bonds provide the self-healing and energy dissipation by reversible bonds breaking, while the integrity of the network is maintained by using strong (“permanent”) dynamic bonds to engineer the mechanical properties of the matrix [13,14].

In this review, we present a summary of the recent research on the design of multifunctional self-healing siloxane materials and strategies that employ advantages of both non-covalent and dynamic covalent bonding in the construction of reprocessable and healable silicone materials with enhanced mechanical properties, designed for various smart materials.

## 2. General Characteristics of Self-Healing Mechanisms in Silicone Elastomers 

**Scheme 1:**
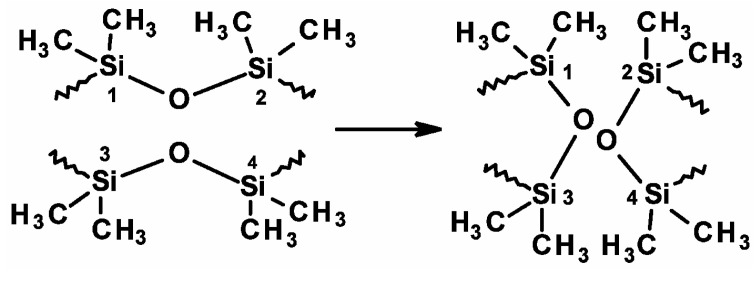
Redistribution of siloxane bonds.

In the case of polysiloxanes with relatively small alkyl substituents at silicon atoms, such as polydimethylsiloxane (PDMS), flexibility of the siloxane backbone allows for fairly easy diffusion of the chains within the sample. As a result, most PDMS elastomers have some ability to self-repair through surface cohesion, but under ambient conditions this mechanism is generally insufficient to fully recover the initial strength of the material. Although it was found that the self-healing mechanism can be activated after damage by anionic equilibrium reactions in crosslinked PDMS (Scheme 1) [15], much more effective self-repair can be achieved with specially designed silicone-based systems.

In general, silicone elastomers can be repaired by extrinsic and intrinsic self-healing processes. The following sections contain characteristics of the different mechanisms. It must be stressed that irrespectively of the type of S-H mechanism and the type of chemical reactions involved in a given system, the effectiveness of self-repairing in siloxane elastomers depends on local dynamics in the matrix (both the dynamic behavior of siloxane bonds and the rate of macromolecules diffusion in bulk material) [5]. Enhanced diffusion accelerates the transfer of healing agents in the polymer matrix towards the damaged area and helps reform broken bonds. Consequently, the defects are typically more easily healed at higher temperatures, whereas the presence of hybrid particles in silicone composites may become a diffusion barrier (increased percolation path and the reduction of cross-section available for migration of macromolecules) and adversely affect the self-healing ability. The S-H process in silicone composites and blends depends on the particles size, concentration, dispersion, and agglomeration in the polymer matrix as well as their interactions with the silicone matrix. In some cases, agglomeration of additives may be beneficial. Interesting S-H effects were reported for silicones with incorporated polyhedral oligosilsesquioxanes (POSS) that self-assembled into well-organized networks [16,17]. POSS-based S-H macromolecular systems have been recently reviewed by our group [18]. In brief, POSS, grafted or embedded in the polymer backbone, can act as active S-H agents (reversible dynamic bond exchange with POSS or the formation of separate inclusions in the polymer matrix through hydrophobic interactions and POSS aggregation). They can also be factors that increase the dynamics of macromolecules in the polymer matrix, and thus facilitate the process of self-regeneration, without being directly involved in the healing mechanism.

### 2.1. Extrinsic S-H of Silicone Elastomers

Early self-repairing silicone materials relied on releasing self-repairing agents into crack interfaces to repair the damaged area (extrinsic self-healing). The S-H approach is constantly developed [19,20] but extrinsic S-H of silicone elastomers is scarcely employed in multifunctional systems that are within the scope of this review. The curing agents in such systems are encapsulated inside micro-vessels dispersed in a polymer matrix. They are released in the regeneration process to fill and seal fractures in the polymer sample as a combination of both physical- and chemical-based self-healing. Extrinsic self-healing of silicones may be based on some specific reactions, such as condensation curing, radical reactions and hydrosilylation, and often involves the use of micro-vesicles containing reactive components [21]. The drawback of extrinsic healing is that they are based on the consumption of pre-embedded healing agents. Thus, the longevity of the effect in this approach is limited by the amount and volume of the capsules. The depletion of reactive agents results in a limited number of healing cycles restricting the durability of the self-healing ability, especially if the same area is damaged.

### 2.2. Intrinsic S-H of Polysiloxane Materials

More complex and efficient systems that can reverse local damage in the polymer matrix are based on processes that involve reversible covalent and non-covalent chemical bonds. Such intrinsic self-healing relies both on the chemical nature of a polymeric material and the polysiloxane chains mobility and entanglements. Due to the requirements of macromolecular dynamics and diffusion, the intrinsic S-H may take place above the polymer’s glass transition temperature (*T*_g_). This process can be enhanced by plasticizing agents, which help lower the effective *T*_g_. The self-healing is achieved by rebuilding the dynamic chemical bonds in the polymer matrix. The regeneration processes can be initiated with light, heat or electric stimuli. Generally, the intrinsic regeneration mechanisms are regarded as more reliable and allow for repeated sample reconstruction.

The types of dynamic bonds include supramolecular interactions (hydrogen bonding, π–π stacking), reversible chemical reactions (Diels–Alder cycloaddition, Schiff base chemistry, disulfide or acyl hydrazone bond formation), and metal–ligand coordination. Crosslinked silicone elastomers can be also constructed with dynamic–covalent boronic esters [22] or host–guest complexes of methylated β-cyclodextrin and adamantane [23]. The use of selected intrinsic S-H processes applied in silicone-based materials chemistry is discussed below.

#### 2.2.1. Polysiloxanes Self-Healed through Reactions of Diels–Alder Cycloaddition

**Scheme 2:**
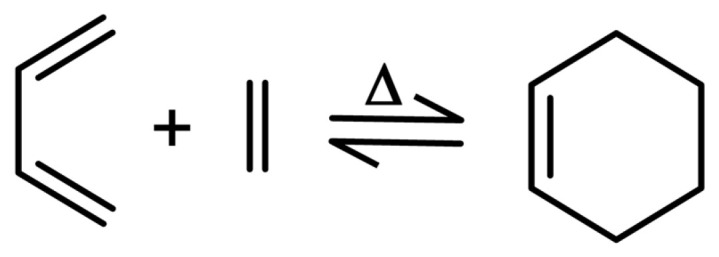
Formation of a Diels–Alder adduct (DA and rDA reactions).

Diels–Alder [4+2] cycloaddition is one of the best-known thermally reversible reactions in organic chemistry [24]. The intrinsic self-healing approach based on the Diels–Alder (DA) and retro-Diels–Alder (rDA) reactions is particularly desirable for the self-repairing of polymeric materials due to its high performance under mild reaction conditions and minimal by-products. The “click” reaction between dienes and dienophiles with the formation of unsaturated six-membered rings (Scheme 2) controls the formation and breaking of bonds under thermal stimuli. It gives crosslinked polymer systems the ability to remold and re-mend.

**Scheme 3:**
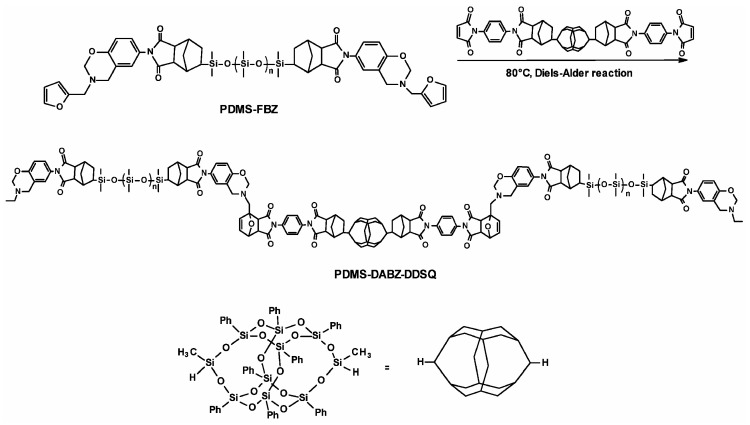
Synthesis of PDMS-DABZ-DDSQ (inset below: chemical structure of a double-decker silsesquioxane cage.

The Diels–Alder reaction was used to design a range of self-healing polysiloxane elastomers. The key was to find suitable precursors that were compatible enough to form a uniform system. The molecular chain mobility and degree of crosslinking must be adjusted as network parameters. DA and rDA reactions in a system composed of polysiloxanes (Mn~13,000 g mol^−1^) grafted with maleimidocarboxyphenyl units (3–4 mol%) and POSS with eight furan moieties (maleimide:furan mole ratio of 1:3.5) occurred at 50 °C and 110 °C, respectively [25]. Bifunctional furan end-grafted PDMS was crosslinked with bismaleimide-functionalized double-decker silsesquioxane (DDSQ-BMI) (Scheme 3) [26]. Thermally stable product (*T*_d10_ = 441 °C; char yield = 60.1 wt.%) of low surface free energy (18.18 mJ m^−2^) was flexible enough (*T*_g_ = 177 °C) to undergo a DA/rDA reaction.

Thermally switchable, hydrophobic adhesives were also prepared using DA-crosslinking between ladder-like poly(silsesquioxanes) (LPSQ) side-grafted with pyrrole and alkyl dienes [27]. The chain mobility was a critical factor for the performance of crosslinked hybrid blends. LPSQ adhesives with long alkyl crosslinkers (LPSQ-Oct and LPSQ-Dec) exhibited the best reversible performance during five repeatable cycles and outstanding softness as well as excellent adhesive strength, surface hardness (up to ~12 MPa) and modulus (up to ~60 MPa) and thermal stability due to the presence of a double silsesquioxane backbone.

Thermo-reversible D-A cycloaddition has been scarcely applied in synergistic S-H of silicone elastomers although hydrogen bonds often play a complementary role. The auxiliary effect was suggested for the crosslinking of silicone copolymers bearing maleimide groups with complementary difuryl siloxane coupling agents (DA at 80 °C and rDA at ~140 °C) [28]. The hydrogen bonding between maleimide carbonyl groups and amide N-H in the telechelic furan linker facilitated the rDA reaction and contributed to improved mechanical properties of the crosslinked silicone elastomer. A tensile strength (*σ*_max_) of 0.61 MPa, ~51% elongation at break ε_max_ and Young’s modulus € of 2.27 MPa were obtained for the optimal network composition. The hybrid elastomer could be thermally reprocessed by hot compression at 140 °C for 1.5 h under a pressure of 20 MPa, followed by 24 h treatment at 80 °C. The healing efficiency *η*_s_ was a function of the polymer structure and the time of healing (29% after 1 h and 95% after 24 h at 80 °C for the optimal composition).

**Scheme 4:**
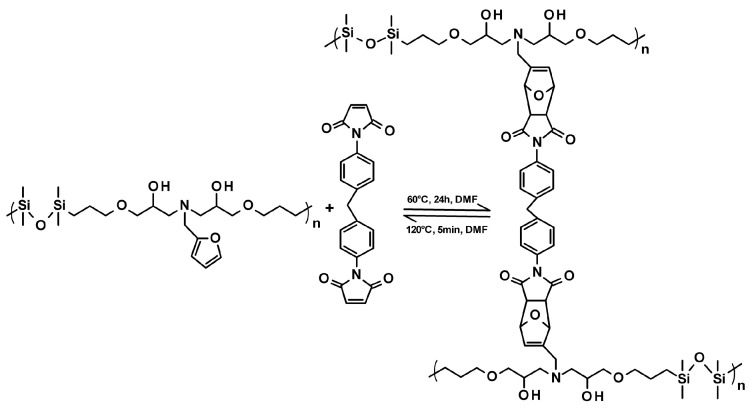
DA crosslinking of PDMS with 4,4′-bismaleimidodiphenylmethane.

DA-coupling was also applied for the crosslinking of polysiloxanes grafted with maleimide groups with silica particles modified with 2-furyl-(undecenyl)-11-triethoxysilane or 3-maleimidopropyltriethoxysilane [29] and ladder-like polysilsesquioxane copolymers and terpolymers, functionalized with both dienophile and diene on the double-stranded siloxane backbone [30]. Furan-2–ylmethylundecanoatesiloxane–dimethylsiloxane copolymers crosslinked with bismaleimide compounds [31] could be repaired in a short time (<5 min) at a relatively low temperature (heat to 80 °C and cool to room temperature to cure). The healed areas were more robust than the unaffected parts. Furan-grafted PDMS copolymers and polysiloxanes crosslinked with 4,4′-bismaleimidodiphenylmethane (BMI) (Scheme 4) [32] were highly adhesive and reprocessable by hot pressing at 150 °C and under 1 MPa for 2 h. The mechanical properties of the regenerated material (strain of 1.05 mm mm^−1^ and a stress of 1.29 MPa) were consistent with the original sample.

Bismaleimide-terminated PDMS and tetra furane functionalized PDMS were thermally cured in the presence of boron nitride (a thermo-conductive filler) [33]. The obtained silicone/BN composite exhibited a high self-healing efficiency (>90%), 544% of thermal conductivity enhancement, 568% increase of tensile strength and a low dielectric dissipation factor of 0.017 at 50 wt.% BN content.

#### 2.2.2. Silicone Self-Healing via Reversible Thiol Coupling Reactions

**Figure 1:**
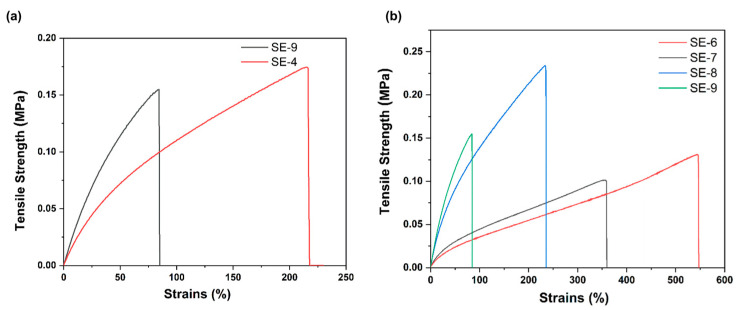
(**a**) Stress–strain curves of SE-4 and SE-9; (**b**) the effect of POSS content on mechanical properties of the elastomer [34].

Self-healing of silicone elastomers through a strategy based on disulphide-linker formation using thiol-functionalized polysiloxanes and thiol-functionalized crosslinkers is an interesting option for self-healing systems [34]. Reversible thiol coupling reactions proceed at room temperature and are accelerated by heating or UV radiation. The structure and amount of the crosslinker plays a pivotal role. A blend (SE-4) prepared with pentaerythritol tetrakis(β-mercaptopropionate) (PETMP) had a higher tensile strength (0.17 MPa) and elongation at break (218%) than its more rigid counterpart (SE-9) crosslinked with octa(3-mercaptopropyl)silsesquioxane (POSS-SH) (respectively, 0.15 MPa and 85%) (Figure 1a). The increase of POSS-SH concentration resulted in a gradual decrease of elongation at break whereas the change in the tensile strength was irregular (Figure 1b). The effect was attributed to phase separation and the formation of POSS-rich and POSS-poor domains of different mechanical properties. Under a thermal treatment (150 °C for 2 h), self-healing efficiency exceeded 70%.

Disulfide bonds chemistry was also involved in the crosslinking of methacrylate/epoxy groups terminated hyperbranched polysiloxanes (HPBSi) that formed hierarchical permanent networks [35]. Hybrid elastomers with reversible dynamic bonds were formed via sequential UV/thermal crosslinking and their mechanical and self-healing performance was adjusted by their composition. HBPSi simultaneously improved the toughness, strength, modulus and thermal stability of hybrid blends. Elastomer (P1-0) without HPBSi was highly stretchable (3000% elongation) but its tensile strength was low (0.17 MPa). The addition of HPBSi improved the tensile strength (0.87 MPa for 5 wt.% and 1.95 MPa for 20 wt.%), yet the limited segmental motion of linear chains resulting from permanent chemical crosslinking reduced the material elongation at break to 241%. Larger concentrations of diaryl disulfide (AFD) linkers resulted in a reduction of tensile strength but better stretchability. The effects of HPBSi and AFD on the mechanical properties corroborated with the S-H performance of crosslinked elastomers that depended on the amount of HPBSi and was improved with the extent of healing time (although the tensile strength was in general decreased after curing). Poor healing ability due to the limited chain mobility was observed at a high concentration of HPBSi. Such dynamically crosslinked polymer, combined with eutectic gallium–indium (EGaIn) conducting alloy, was applied as a flexible substrate in smart wearable electronic systems (motion sensors) [35].

**Figure 2:**
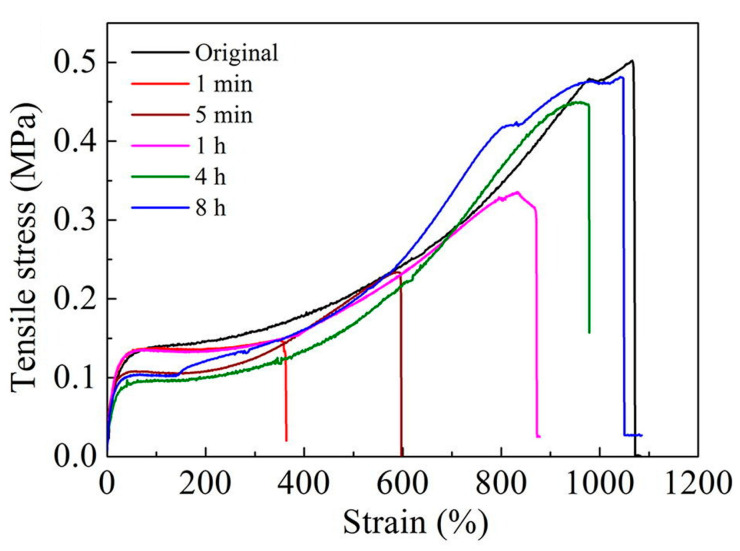
Stress–strain curves of an AFD-containing silicone elastomer healed at room temperature for different times. Reprinted from [36], Copyright (2020), with permission from Elsevier.

Positive effects of the presence of aromatic disulfides on tunable stretchability of siloxane matrix and its rapid self-healing by room temperature metathesis of S-S bonds were also observed in similar systems [36]. The effect can be tuned by the crosslinking density (amount of AFD), the molecular weight of siloxane and the stretching rate. For some AFD concentrations, sample elongation over 2500% or the ultimate tensile stress of 1.216 MPa could be achieved (not simultaneously). A sample of optimal composition exhibited an elongation at break over 1000% and a tensile stress of 0.5 MPa. This elastomer could efficiently dissipate the strain energy and its S-H efficiency exceeded 95% at room temperature, even after surface aging. Healing over a very short time (1 min) resulted in almost 30% of the original mechanical properties (Figure 2). The elastomer was employed as a substrate for self-healing stretchable electronics.

#### 2.2.3. Self-Healing of Silicones Using Schiff Base Chemistry

**Scheme 5:**
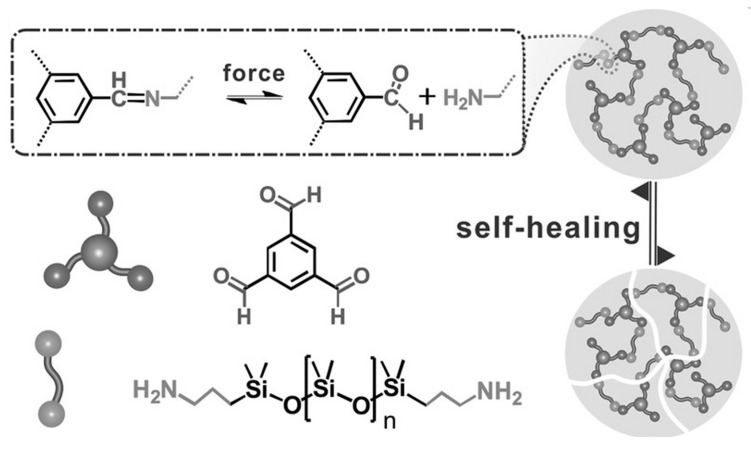
Self-healing of silicone elastomer by action of dynamic covalent imine bonds. Adapted from [40], Copyright (2017), with permission from John Wiley & Sons, Inc.

Schiff bases are worth noting in S-H systems because of their dual action. They can be excellent ligands for the coordination of metals due to the presence of a lone electron pair at nitrogen atoms [37]. Various Schiff base/metal complexes were applied to improve mechanical properties of silicone elastomers by reversible crosslinking [38]. Schiff bases can also be an interesting source of dynamic covalent bonding [39]. Imine bonds formed in the Schiff base reaction between telechelic amine-terminated PDMS elastomer and 1,3,5-triformylbenzene (TFB) acted as dynamic crosslinking sites reversible under mild conditions (Scheme 5) [40]. The zwitterionic intermediate was stabilized because of thr electron-accepting action of the aromatic ring.

Transparent stretchable networks (ε_max_ up to ~700% at 5 mm min^−1^ stretching rate) were formed. Mechanical properties depended on the molecular weight of the PDMS chain, the stoichiometry of the composition and the rate of stretching. A shorter time for the reformation of imine bonds at high stretching rates reduced the fracture tolerance. The waiting time (no-contact) had a negligible effect on *η*_s_. A very short contact was sufficient for effective self-healing of the Schiff base silicone networks (53% *η*_s_ after 1′ at room temperature; 100% within 60′) and re-mending was accelerated by heating (100% *η*_s_ after 30′ at 70 °C). The hydrophobic hybrid elastomers were self-healable in a wide range of conditions, including low temperatures (−20 °C in air) and an aqueous environment. It makes them useful as anticorrosion coatings, adhesives, encapsulants and flexible interconnectors.

#### 2.2.4. Silicone Elastomers’ Autoregeneration through Acid–Base Ionic Interactions

Self-healing in silicone elastomers may occur also through acid–base interactions. Supramolecular S-H silicone networks were also built by crosslinking siloxane oligomers with a low content of amino (6.4 or 9.8 mol%), and carboxyl (14.9 mol%) groups [41]. Thermal, mechanical and electrical properties of such elastomers can be adjusted by the content and the ratio between the two types of polar groups and the morphology of the siloxane oligomer.

**Scheme 6:**
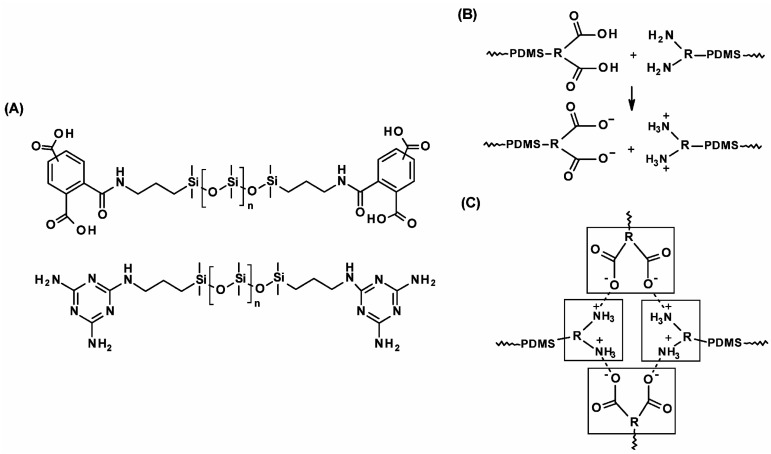
Chemical structure of functionalized telechelic siloxane oligomers (**A**) and ionic interactions they can be involved with (**B**,**C**).

Ionic self-healing with the formation of ammonium carboxylate groups also occurred in supramolecular ionic blends obtained by mixing telechelic PDMS oligomers terminated with amine and carboxyl groups (Scheme 6) [42].

The values of tensile modulus (*E*), tensile strength (*σ*_max_), elongation at break (ε_max_), for uncut samples, and strength healing efficiency (*η*_s_) depended on the length of PDMS chains. The ionic crosslinking density increased when short-chain oligomers were applied, resulting in the formation of a more stable blend with a predominant elastic behavior at a higher temperature. The effect of the type of the chain end was noticed in tri-component blends where the glass transition was shifted to a higher temperature range, because of the rigidity of the base chain-ends. *E* of ~10,500 kPa and *σ*_max_ of ~700 kPa, ε_max_ of 9% and 25% *η*_s_ were obtained for the rigid system, whereas the introduction of longer PDMS chains resulted in a more stretchable network (*E*~850 kPa, *σ*_max_ 215 kPa, ε_max_ of 43% and 27% *η*_s_).

#### 2.2.5. Silicone Elastomers Self-Healed by Means of Hydrogen Bonding

**Scheme 7:**
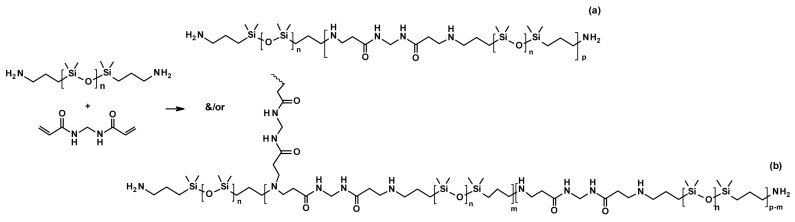
Linear (**a**) and branched (**b**) products obtained by a step growth process based on aza-Michael addition of bis(3-aminopropyl)-terminated PDMS to N,N′-methylenebis(acrylamide).

Supramolecular interactions based on hydrogen bonds are most often applied in the tunable S-H of siloxane elastomers as their strength and effectiveness depends on the structure of bonded segments (e.g., urea–urea, urethane–urea and urethane–urethane groups, in the order of strength [43]). Such systems quite often demonstrate phase-separated morphologies and an effective migration of chain segments is required for an efficient exchange of dynamic hydrogen bonds. Linear and branched copolymeric silicone elastomers with self-associating bis(β-aminoamide) groups (Scheme 7) can be an example [44]. The phase separation due to H-bond interactions between amide groups was reflected in two thermal transitions: *T*_g_ of PDMS block (−123 °C) and dynamic redistribution of hydrogen bonds in the organic domains (a broad endothermic peak at around −40 °C). The crosslinking through H-bonding and entanglement of macromolecules improved tensile properties of the elastomer (~0.1 MPa ultimate tensile strength and ~1500% strain at break). The self-healing capability (the mechanical parameters were recovered after S-H at room temperature for 72 h) was also related to the combined action of hydrogen bonds and short chain diffusion.

**Figure 3:**
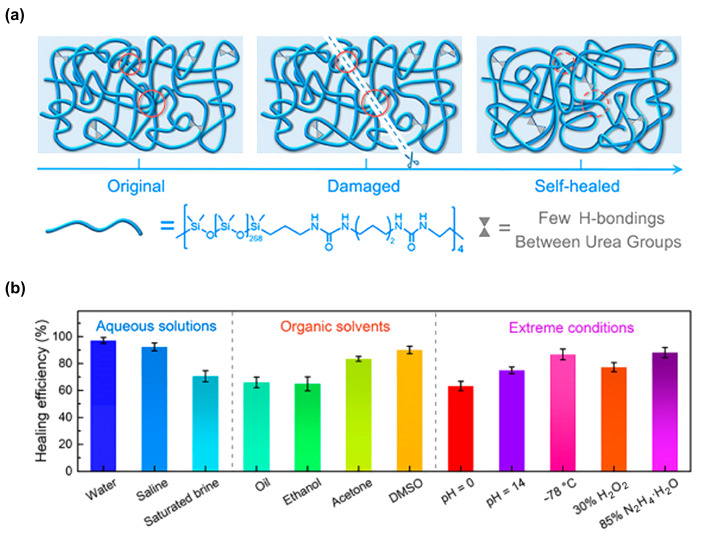
(**a**) Schematic self-healing process driven by polymer chain diffusion and entanglement (red circles indicate the sites of chain entanglements redistribution); (**b**) self-healing efficiencies of the polymer determined by the corresponding tensile plots. Adapted with permission from [45]. Copyright (2021) American Chemical Society.

The urea segments (-HN-CO-NH-), generated by coupling isocyanate- and amine-functionalized monomers, play a very important role in S-H mechanisms. They are capable of thermally reversible, strong intermolecular hydrogen bonds which endows silicone elastomers with self-healing properties and can significantly improve their stretchability. The physical entanglements in PDMS–elastomers with urea groups in the main chains played a significant role in their autonomous self-healing [45]. It was achieved without any external stimulus in various environments, including extreme conditions (Figure 3). 30 min of healing was sufficient to regain the original polymer stretchability.

The polymers were thermally stable up to 410 °C under an air and N_2_ atmosphere. Their mechanical performance during uniaxial stretching tests depended on the stretching rate. Recovery of the original energy dissipation capacity, long-term durability and fatigue resistance were displayed in cyclic tensile tests. It was also shown that the S-H polymer can be integrated with a eutectic gallium–indium (EGaIn) alloy to achieve a layer-by-layer self-healing soft electronic (e.g., skin sensors for harsh environment). 

**Scheme 8:**
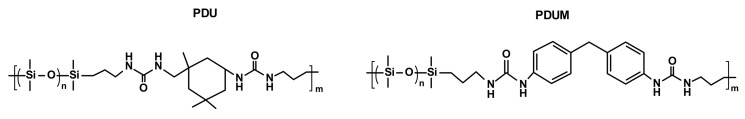
Molecular structures of urea silicones used for preparation of S-H coatings.

An optimal balance between polymer chain flexibility and diffusion, and hydrogen bonding site density is crucial for the efficient self-healing of siloxane elastomers. This approach was used in the case of transparent and recyclable ice-phobic polydimethylsiloxane–urea coatings (Scheme 8) [46]. They exhibited an ultrafast self-healing capability (80% of the ultimate tensile strength *σ*_max_ was regained within 45′ at room temperature). The ice adhesion was low (~50 kPa during 20 icing/de-icing cycles), even after mechanical damage. Such polymers are suitable for icing protection on surfaces that require high light permeation for operation (e.g., solar panels and sensors).

**Scheme 9:**
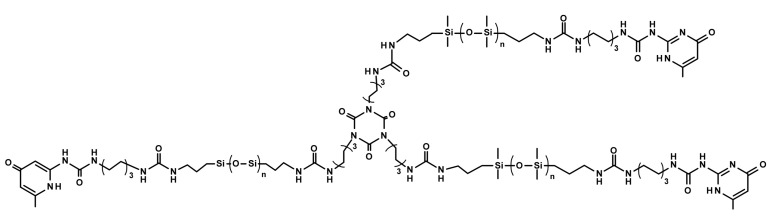
Star-shape structure of PDMS with embedded urea segments.

The urea derivatives can also form hydrogen bonds to metal oxides. A siloxane copolymer of star-like topology and embedded urea segments (Scheme 9) displayed self-healing properties and outstanding adhesion to NiO [47]. These properties made the copolymer a promising candidate as a polymer matrix of thermal interface materials (TIM).

A high density of hydrogen-bonded segments in these macromolecules resulted in enhanced inter-segment interactions and strength in uniaxial tensile tests. The polymer was most ductile at 102 °C but 177 °C was found to be the optimal temperature for self-healing (*η*_s_ = 85%). The dependence of mechanical properties on the tensile rate was correlated with differences in the relaxation of H-bonded and non-bonded units. The nonbonding interactions were influenced most at fast tensile rates (insufficient relaxation time) whereas the effect of bonding interactions was gradually enhanced with the increase of tensile rate.

**Scheme 10:**
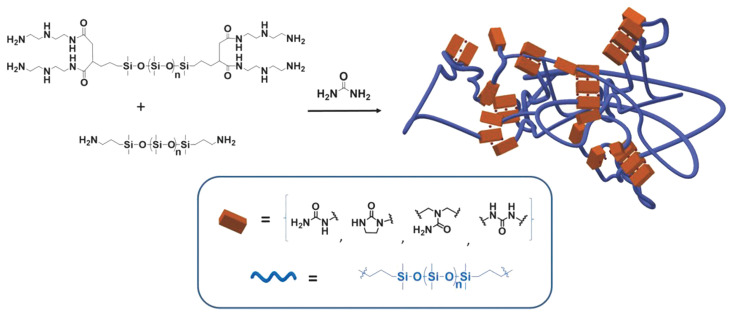
Crosslinked PDMS elastomer networks by formation of urethane segments. Reprinted from [48], Copyright (2018), with permission from John Wiley & Sons, Inc.

**Figure 4:**
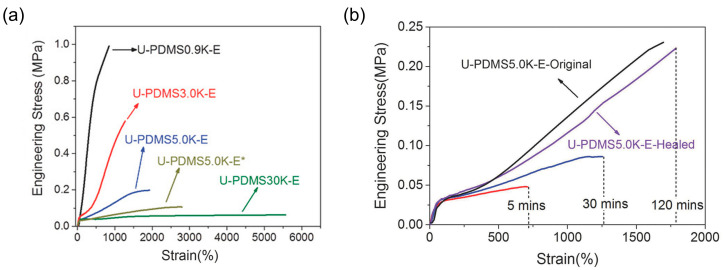
Strain–stress curves of PDMS-U networks (**a**) with polysiloxane segments of different molecular weight (molar ratio of substrates (tetra acid terminated PDMS:amine-terminated PDMS) is fixed at 1:5 except for U-PDMS-E* (1:10), (strain rate 1 s^−1^) and (**b**) the effect of self-healing at 25 °C with time. Adapted from [48], Copyright (2018), with permission from John Wiley & Sons, Inc.

Another self-healable network obtained with urea and amine-functionalized PDMS exhibited high stretchability and recoverable gas-separation performance (Scheme 10) [48]. The mechanical properties of the networks could be tuned through the ratio of components and molecular weight of rubbery PDMS segment. The networks exhibited rubber-like properties and very high elongation at break (984–5600%, the elasticity was strain-dependent) (Figure 4) as well as acoustic and vibration damping properties over temperature −40 °C–+100 °C. Their elastic recovery depended on the length of PDMS chains (better results were achieved for shorter oligomers). After mechanical damage, the networks healed completely in 120′ at room temperature (or in 20′ at 40 °C). The mechanical performance (Young’s modulus, extensibility, ultimate tensile strength and toughness) and gas-separation properties were completely restored.

**Scheme 11:**
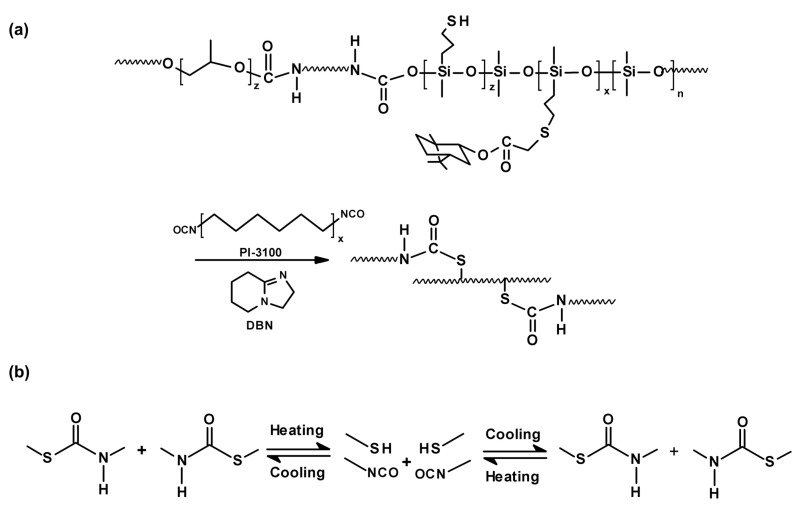
Synthesis of poly(thiourethane-urethane) (**a**) and mechanism of reversible thiourethane exchange (**b**).

Silicone elastomers can be also crosslinked by means of dynamic covalent thiouretane bonds. Their activation energy (125 kJ/mol [49]) is promising for efficient self-healing providing suitable mobility of polymer chains. Consequently, hydrophobic polysiloxanes with side isobornyl acrylate and thiol groups (Scheme 11) exhibited good mechanical performance and could be reprocessed and recycled at 150 °C [49].

The sterically hindered isobornyl acrylate groups promoted siloxane segments’ migration and thus accelerated the exchange of thiocarbamate bonds, which was reflected in the rate of the regeneration process. A scratch in the film disappeared after a 1 day treatment at 90 °C. The mechanical performances of the hybrid elastomers were also significantly increased due to the synergistic effect of rigidity and crosslinking. The high flexibility of polysiloxane segments improved their elongation at break. The tensile strength *σ*_max_ of the film increased from 4.70 to 12.56 MPa on the introduction of isobornyl substituents.

*Silicones Crosslinked with Multiple Paired Hydrogen Bonds*

**Scheme 12:**
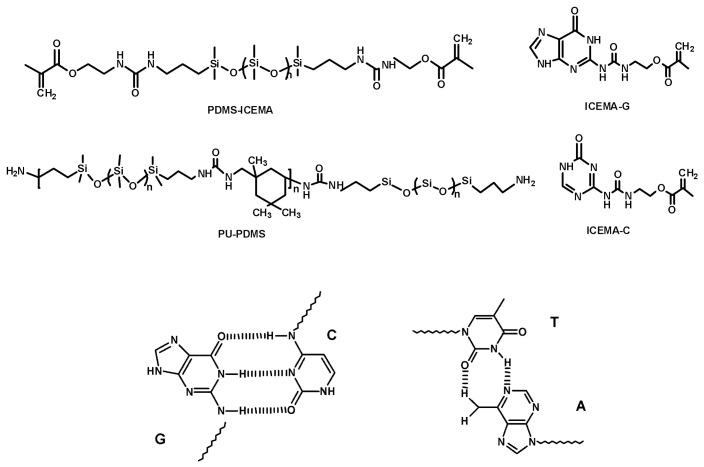
Chemical structure of PDMS elastomers used for the preparation of networks crosslinked by nucleobase recognition (below the structure of the hydrogen-bonded nucleobase pairs G-C and A-T).

The formation of multiple hydrogen bonds can provide dynamic homo- and hetero-assembly of functionalized siloxane chains. Supramolecular crosslinking of this type was employed in the case of silicone elastomers functionalized with nucleobases—adenine (A) and thymine (T) [50]. A–A, T–T and A–T base pairs related recognition governed the mechanical properties of the material and was tuned by adjustment of the nucleobase type and content. The A–T hetero-pair (Scheme 12) elastomers displayed better mechanical properties than their A–A or T–T homo-assembly analogues. The elastomers exhibited ultra-low temperature (−114.5 °C) resistance because the formation of hydrogen-bonded base pairs limited the movement of the polysiloxane chains. Analogously, silicones with nucleobase pairs—guanine and cytosine (G-G, C-C, G-C) linked by three hydrogen bonds (Scheme 12)—showed good self-healing efficiency, elasticity and recoverable mechanical performance (tensile strength) [51]. 

**Figure 5:**
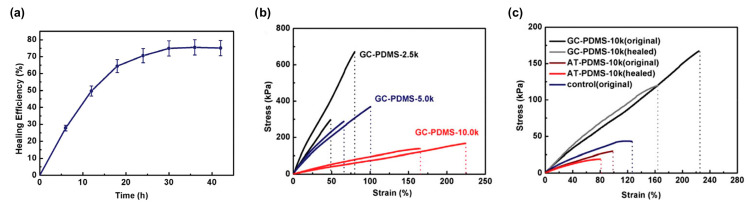
(**a**) Changes of self-healing efficiencies with time of treatment at 80 °C and tensile tests obtained for original and healed GC-PDMS of different lengths of polysiloxane block (**b**) and different base pairs (**c**). Adapted from [51], Copyright (2019), with permission from John Wiley & Sons, Inc.

Their self-healing efficiency and the mechanical performance depend on the number of hydrogen bonds between base pairs (Figure 5). It was postulated that the steric hindrance of amino groups in guanine and cytosine might also affect the strength of hydrogen bonds. The self-healing ability and elasticity of the hybrid material could be adjusted on changing the molecular weights of the PDMS segment (Figure 5b). The strain–stress curves illustrated an enhancement of ductility on the increase of length of PDMS blocks and a deterioration of both ultimate strength and strain at breaking when the G-C pair was changed for A-T nucleobases (Figure 5c). The self-healing efficiency of G-C-PDMS was a function of healing time and temperature (optimal healing T = 80 °C after ~30 h). Prolonged heating at 90 °C caused a partial degradation of the polymer.

A specific group of self-healing silicone elastomers contains ureidopyrimidinone (UPY) units that can form unique dimeric motifs and direct organization of silicone macromolecules into a 3D network. Silicones crosslinked by UPY display outstanding properties that can be related both to high association energy of quadruple hydrogen bonds and microphase separation. Silicone elastomers with UPY groups in their side chains displayed an excellent mechanical performance (stiffness~272 MPa; toughness~8.0 MJ·m^−3^), strong interfacial adhesion (up to ~9.0 Mpa) to various substrates (stainless steel, aluminium, copper, epoxy and glass) and good reusability [52].

**Scheme 13:**
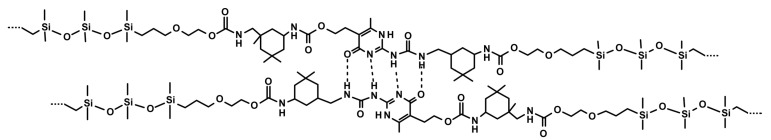
Synthesis of UPY-containing poly(siloxane–urethane) and their crosslinking by the quadruple hydrogen bonding.

Polysiloxanes with UPY in the main chains were prepared by co-polycondensation of hydroxyl-terminated PDMS (HO–PDMS–OH), isophorone diisocyanate (IPDI), and 5-(2-hydroxyethyl)-6-methyl-2-aminouracil (HMA-UPY) (Scheme 13) [53]. This approach enabled a well-defined spacing length between UPY groups and control over the polymer network structure.

**Figure 6:**
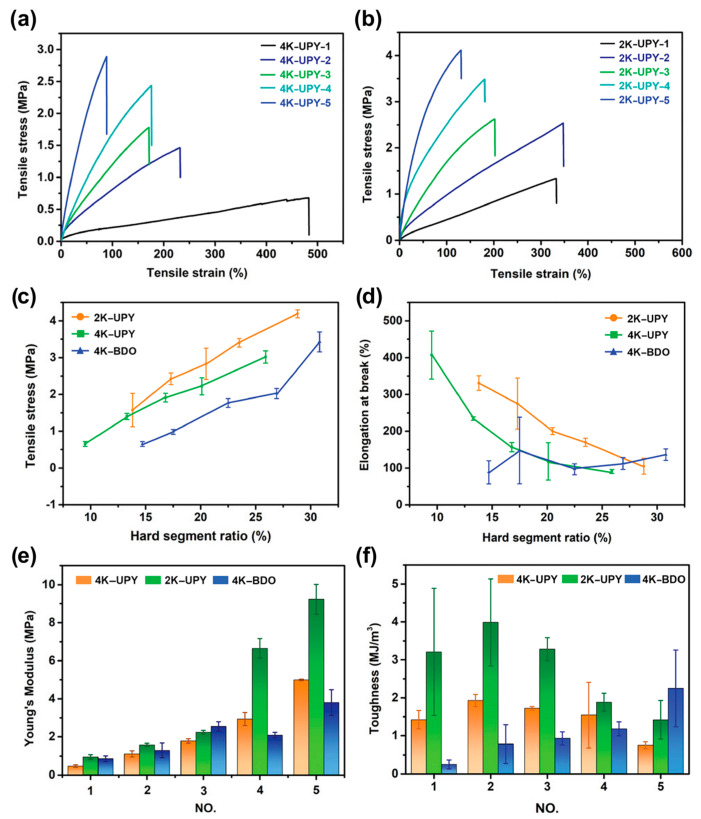
Comparative mechanical characteristics of linear PDMS-UPY elastomers: (**a**) tensile tests of (**a**) PDMS-4K-UPY and (**b**) PDMS-2K-UPY; comparisons of (**c**) tensile stress, (**d**) elongation at break, (**e**) Young’s modulus, and (**f**) toughness of PDMS-4K-UPY, PDMS-2K-UPY and PDMS-4K-BDO elastomer. Reprinted from [53], Copyright (2021), with permission from John Wiley & Sons, Inc.

Comparative tests showed that the multivalent hydrogen bonding of UPY was responsible for the observed enhancement of tensile properties (Figure 6). The content of soft and hard segments adjusted by changes in the ratio of PDMS to UPY or the length of siloxane chains was reflected in mechanical properties of the hybrid materials. The reversible quadruple hydrogen bonds made the elastomers reprocessable both through solution treatment and by hot compression.

Moreover, the PDMS-UPY could be developed into water-responsive luminescent metallo-supramolecular elastomers by the coordination of europium ions to UPY groups. The pristine elastomer emitted blue luminescence at ~425 nm on irradiation with UV light. The effect was attributed to the aggregation of electron-rich heteroatoms resulting in the suppression of nonradiative energy loss. The incorporation of Eu^3+^ changed the elastomer’s emission wavelength to red (emission peaks at 590, 616 and 693 nm) due to 5D_0_ → 7F_1_, 5D_0_ → 7F_2_ and 5D_0_ → 7F_4_ transitions of Eu^3+^ ions. In addition, the elastomer exhibited a reversible luminescence quenching in contact with water (the photoluminescent color gradually changed from red to purple because of the competitive coordination of water molecules). The red color was restored after H_2_O evaporation at 60 °C.

**Scheme 14:**
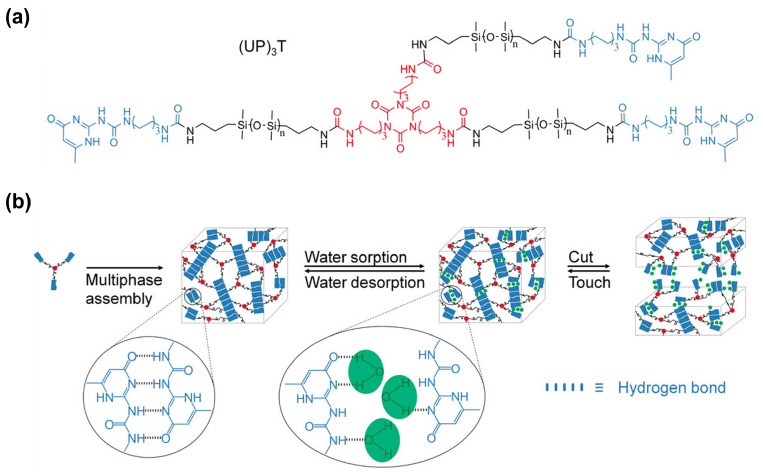
Chemical structure of (UP)_3_T macromolecules (**a**) and multiphase design of their multiphase assembly and self-healing process in the presence of H_2_O (**b**). Reprinted from [12], Copyright (2018), with permission from John Wiley & Sons, Inc.

Multivalent hydrogen bonding between UPY groups was also employed in multiphase-separated silicone 3D networks constructed with star-shaped siloxane macromolecules (UP)_3_T [12]. (UP)_3_T was synthesized from a tri-functional homopolymer of hexamethylene diisocyanate, amino-terminated PDMS and UPY chain terminating groups (Scheme 14). The hydrogen-bonded UPY formed hard stacks in the soft and hydrophobic siloxane matrix which resulted in the formation of a microphase-separated, semi-crystalline polymer network of high mechanical strength. Tensile tests showed that the as-prepared material is strong and stiff, with a high Young’s modulus (~47.4 MPa) and storage modulus (~151 MPa by DMA) measured at room temperature. It can be related to the high density of 3D crosslinking with UPY dimers and stacks placed among hydrophobic polysiloxane chains. The thermoplastic supramolecular polymers were readily melt-processed into transparent solids almost not losing their mechanical properties.

**Figure 7:**
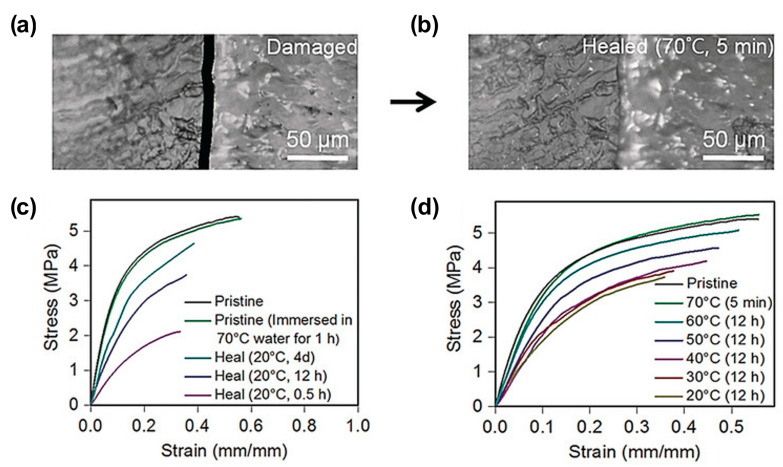
Healing of (UP)_3_T films: optical microscopic image of a damaged film before (**a**) and after healing in water bath (70 °C) for 5 min (**b**), and tensile stress–strain curves of (UP)_3_T healed in water (**c**) at 20 °C for different periods of time or (**d**) for 12 h at various temperatures (5 min at 70 °C). Reprinted from [12], Copyright (2018), with permission from John Wiley & Sons, Inc.

Interestingly, the self-healing of these materials turned out to be enhanced by water (S-H underwater: 70 °C, 5 min, 98% recovery of strength) (Figure 7). It was attributed to penetration of the hydrophobic polysiloxane matrix with H_2_O molecules that, dissociating the multivalent hydrogen bonds in UPY-rich microphase domains, acted as a plasticizer. The healing efficiency increased with the relative humidity, healing temperature and healing time.

## 3. Silicone Elastomers Acting through Multiple Synergistic Self-Healing Mechanisms

Smart adaptive materials require polymer matrices with high elasticity and regenerative capacity. The flexibility of the polysiloxane backbone gives the material softness and elasticity. Dynamic covalent chemistry combined with non-covalent interactions provides synthetic methodologies for the synthesis of self-repairing materials. Modifying the length of the siloxane chains provides a range of mechanical properties that can be tuned by the inclusion of hybrid components (organic units with covalent dynamic bonds or supramolecular interactions). The mechanical properties of the system can also be tuned by changing the ratio of strong and weak hydrogen bonds.

The self-healing effect in such systems is the synergistic result of multiple interactions in supramolecular polymer networks. Thus, the properties of functional materials can be conveniently tuned by modulating their molecular structure. In the following sections, we present characteristics of such systems with multiple dynamic self-healing mechanisms.

### 3.1. Multifunctional Elastomers with Urea Motifs Incorporated as Hydrogen Bonding Sites

#### 3.1.1. Silicone-Urea Self-Healing by Combined Action of Various Hydrogen Bonds

The self-healing potential and toughness of PDMS-based elastomers can be improved by a combination of advantages of different reversible hydrogen bonds and formation of phase-separated morphologies. H-bonds of various strengths may bring in multiple response mechanisms to the elastomeric network. Strong interactions, especially those that can form hierarchical structures within H-bond rich domains, maintain integrity of the crosslinked elastomeric network and provide rigidity and strength to the elastomer. Weak hydrogen bonds can serve as sacrificial linkages that take part in the dissipation of mechanical energy during deformation.

For example, linear carboxyl-terminated polydimethylsiloxane oligomers, diethylenetriamine (DETA) and urea were used as precursors of hybrid polymers with 1,1-dialkylurea motifs [54]. They were crosslinked into amorphous, supramolecular elastomers by H-bonding between 1,1-dialkylurea groups and imidazolidone derivatives. Four stages of the network evolution were postulated that involved first the formation of an associated liquid, then a viscoelastic fluid, unstable viscoelastic solid and finally stable elastic material. These stages were reflected by changes in physical properties and the increasing mechanical strength with increased content of bonded groups. The rheological, mechanical and self-healing properties were dependent on the length of PDMS oligomers and the viscoelastic properties were dependent on the chains entanglement in the amorphous matrix. Unfavorable distribution of the hydrogen bond types formed between different organic groups resulted in poor stacking behavior. Only hydrogen bonding between 1,1-dialkylurea motifs and imidazolidone derivatives were effective enough to contribute to the stability of the supramolecular networks. The temperatures required for S-H were related to the PDMS chain lengths. The shorter oligomers, the higher temperatures and longer healing times were necessary to achieve suitable mobility of chains and non-associated groups on the fracture surface.

**Scheme 15:**
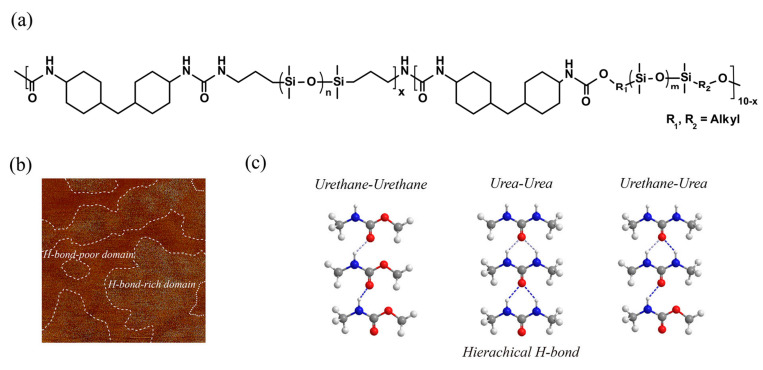
(**a**) Chemical structure of the hybrid elastomers; (**b**) AFM image of a phase-separated morphology; (**c**) hierarchical H bonds formed in elastomers between urethane–urethane, urea–urea and urethane–urea moieties (●—N, ●—O, ●—C, ●—H atoms). Adapted with permission from [55]. Copyright (2020) American Chemical Society.

A super tough (up to 24,000 J/m^2^), self-healable notch-insensitive and transparent siloxane elastomer (PDMAS-U10) (Scheme 15) was prepared following a similar approach [55]. The hybrid elastomers were characterized by a phase-separated morphology due to the differences in the non-polar PDMS segments and polar H-bonded urea units. Hydrogen-bonded urea units formed small-size separate domains that acted as crosslinking sites. The phase-separated material displayed two characteristic loss modulus (G″) peaks in DMA traces, that were ascribed to the glass transition of PDMS segment and association/dissociation of hydrogen bonds.

Both phase-separated morphology with domains rich either in flexible PDMS chains or H-bonded segments and dissociation/association of hierarchical hydrogen bonds make the elastomer self-healable, highly stretchable and having high fracture energy. The mechanical properties of the hybrid macromolecules were robust (tensile strengths *σ*_max_ of 0.98 and 2.17 MPa, and Young’s moduli of 0.75 and 1.03 MPa, depending on the polymer block composition). Uniaxial stretching tests demonstrated their low sensitivity to stretch speed, indicating a quick recovery of the broken hydrogen bonds. The same factors influenced the self-healing efficiency that was found to be highly dependent on the elastomers’ composition and temperature. Samples containing larger amounts of H-bonded structures healed better at higher temperatures, when the polymer chain motion increased. PDMAS-U10 was highly adhesive with hydrophobic PDMS and was applied for the encapsulation of poly(2-acrylamido-2-methyl-1-propanesulfonic acid) (PAMPS-U10). It was exploited for the preparation of a transparent triboelectric nanogenerator (TENG) that was fabricated by sandwiching the PDMAS-U10/PAMPS-U10 as the conductive layer, between two pieces of the PDMS elastomer. The constructed TENG was tough (16,500 J/m^2^), self-healable (*η*_s_~97%) and transparent (>87%).

**Scheme 16:**
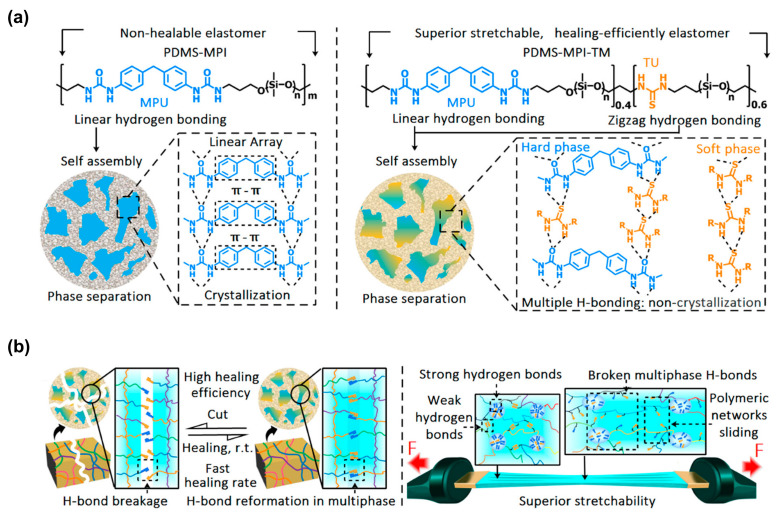
(**a**) Chemical structure of PDMS-MPI-TM and interactions in the phase-separated domains, (**b**) Schematic illustration of the proposed mechanism for healing after damage (left) and hydrogen bonds rearrangement during stretching (right). Reprinted with permission from [56]. Copyright (2019) American Chemical Society.

The self-healing efficiency of silicone–urea elastomers may be also tuned by the introduction of thiourea motifs. Thiourea groups were incorporated as an anti-crystallization agent into a microphase-separated PDMS-based polyurea network crosslinked with multi-strength H-bonds (PDMS-MPI-TM) (Scheme 16) [56].

The presence of dynamic reversible H-bonds in both hard and soft segments resulted in a very good self-healing performance. The polymer was ultra-stretchable (up to 31,500% without fracture) and notch-insensitive (stretching up to 18,000%) because of the multiphase H-bonds. The elastomer recovered its mechanical properties within 4 h at room temperature. The S-H process also took place at −20 °C (85% efficiency after 48 h) or in water (95% efficiency after 4 h). Those features make it suitable for the design of highly stretchable self-healable underwater operating devices.

**Scheme 17:**
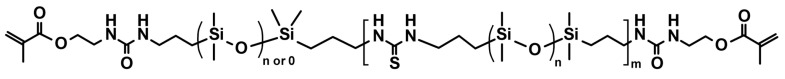
Chemical structure of a methacrylate-terminated PDMS with thiourea motifs.

The reduction of unfavorable crystallization risk in urea–thiourea silicones due to the geometrical nonlinearity of H-bonded thiourea arrays enabled the application of self-healing hybrid silicone elastomers as resins usable in conventional digital 3D printers. Methacrylate-terminated PDMS-urethane with thiourea segments in the main chain (Scheme 17) were found to be suitable as photoresins for stereolithography-based high resolution printing [57]. 

Microstructured objects of an excellent elasticity were prepared, with a potential for application in medical devices, wearable electronics and soft robotics. The improved intermolecular interactions in the amorphous silicone elastomers enhanced the mechanical properties while maintaining a relatively low viscosity of the resin. The cured silicone elastomer of optimal composition exhibited up to ~1000% elongation under tensile load and good cyclic compression durability.

#### 3.1.2. Polymeric Materials Self-Healing by Combined Action of H-Bonded Urea Groups and Dynamic Disulfide Bonds

Hydrogen bonding between soft and hard blocks in hybrid PDMS elastomer in cooperation with dynamic disulfide bonds can be a means for dynamic microphase separation in S-H silicone elastomers. This approach may bring in an improved toughness and tensile properties, much required in the case of, e.g., wearable electronic devices. An example can be a transparent hybrid polysiloxane elastomer of high tensile strength (*σ*_max_ 1.89–3.33 MPa), high stretchability (ε_max_ 347–1722%) and fracture toughness of 28.6 MJ m^−3^ [58]. The low energy S-S bonds were locked mostly in the hard phase, but they could be activated at temperatures > *T*_g_ resulting in self-healing (S-H efficiency > 90% at 80 °C) and good recyclability of the system.

**Scheme 18:**
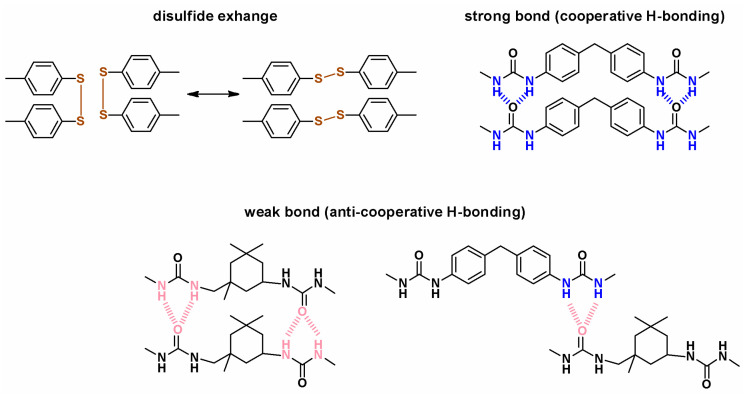
Chemical structure of different type dynamic bonds applied for crosslinking of siloxane elastomers.

Polysiloxanes crosslinked by hydrogen bonds of various strength (strong cooperative H-bonds between aromatic urea segments and weak anti-cooperative H-bonds between alkyl urea and alkyl/aromatic urea segments) combined with the presence of dynamic disulfide bonds (Scheme 18) were designed as recyclable elastomers that can be reformed by hot pressing or dissolution [59].

Their self-healing capacity (*η*_s_ = 75%), good mechanical strength (*σ*_max_~4.67 MPa and ~27.36 MJ m^−3^ toughness) and high stretchability (tensile strain ε_max_ of ~1086%) was fine-tuned by the adjustment of the ratio of different strength hydrogen-bonded sections and the amount of aromatic disulfides. It was also shown that the hybrid material can be used for encapsulation of metal alloy (EGaIn) particles that can be exploited for applications in smart wear and pressure/motion sensors [59].

**Scheme 19:**
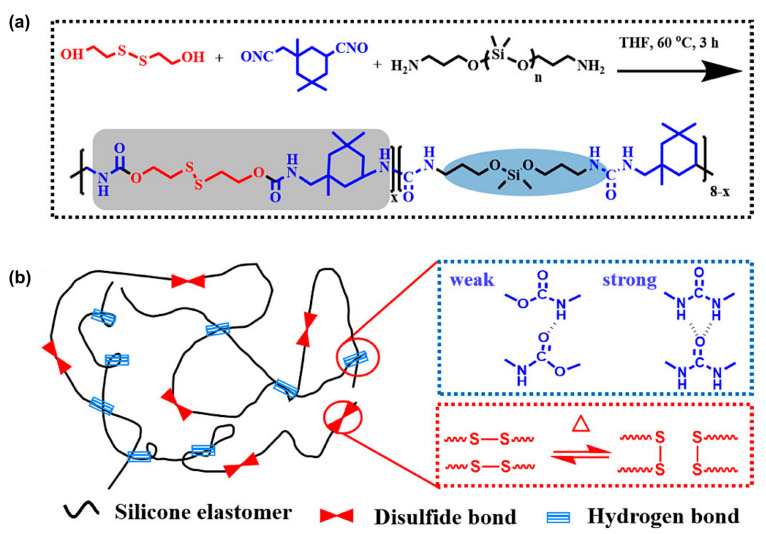
Synthesis of self-healing silicone elastomers (**a**) and schematic diagram of interactions in the elastomer network structure (**b**). Reprinted with permission from [60]. Copyright (2021) American Chemical Society.

Moreover, supramolecular S-H silicone elastomers crosslinked by a combination of multiple dynamic disulfide bonds and H-bonding between urea and urethane groups were designed for potential application as electrothermally driven artificial muscles [60]. Synthetic materials of this type require a fast response and capability of large tensile and torsional actuations as well as durability or self-regeneration. A one-pot *co*-polycondensation of bis(3–aminopropyl)–terminated poly(dimethylsiloxane) (Mn = 1800 g mol^−1^)/isophorone diisocyanate (IP)/2,2′-diethanol disulfide (HEDS) resulted in three types of silicone elastomers (Scheme 19). The monomer ratios of 6:8:2 resulted in sample IP-HEDS2. Silicone elastomers IP-HPSX and IP-APSX were obtained by replacing HEDS with bis(4-hydroxyphenyl) disulfide (HPS), and 2,2′-diaminodiphenyl disulfide (APS), respectively. The degree of microphase separation gradually increased in the order of IP-HEDS_2_ < IP-HPS_2_ < IP-APS_2_ which corroborates the increase of π–π stacking interactions and hydrogen bond strength. 

The elastomers exhibited excellent mechanical properties (antipuncture, stretchability and notch-insensitivity). The stronger notch-insensitivity of IP-HPS_2_ can be also attributed to hierarchical hydrogen bonding interactions of different strengths. Strong hydrogen bonds between urea groups prevent strain-induced crack diffusion, while breaking weaker urethane H-bonds and disulfide bonds help dissipate strain energy. Elongation at break (ε_max_) gradually decreased with an increase in S-S bonds content (ε_max_ of 1504% was obtained for IP-HEDS_2_). The maximum stress (*σ*_max_) and elastic moduli (*E*) of the three kinds of silicone elastomers depended on the elastomers composition. Larger *σ*_max_ values were noted for samples containing hard segment regions, enriched in physical crosslinks formed by hydrogen bonds, disulfide bonds and π–π stacking interactions (*σ*_max_ of 6.1 MPa for IP-APS_3_ and *E*_max_ of 19.2 MPa for IP-APS_4_). The effective energy dissipation by dynamic non-covalent bond reversible breaking was indicated by hysteresis in cycle loading–unloading at a maximum tensile strain of 200%.

A good balance between dynamic bonds and hard segment content was required for effective self-regeneration. The self-healing ability of these elastomers depended on their composition and increased with the duration of healing at 40 °C, reaching, e.g., 98.4% after 24 h for IP-HEDS_5_. Elastomers IP-HPS*_X_* and IP-APS*_X_* required heating for an effective self-repair; respectively, 100 °C in the case of IP-HPS*_X_*, and 120 °C for IP-APS*_X_*. The S-H efficiency of IP-HEDS*_X_* increased with the increase of disulfide content. On the other hand, IP-APS_4_ and IP-APS_5_ with higher hard segment contents that hindered the movement of molecular chains and prevented their effective diffusion and reconnection of dynamic bonds did not heal even at 120 °C. The results were supported by *T*_g_ trends obtained with DMA measurements. It was also shown that the metathesis of dynamic disulfide can be applied for self-healing between the different types of silicone elastomers (e.g., mixtures of IP-HEDS*_X_* and IP-HPS*_X_*).

**Scheme 20:**
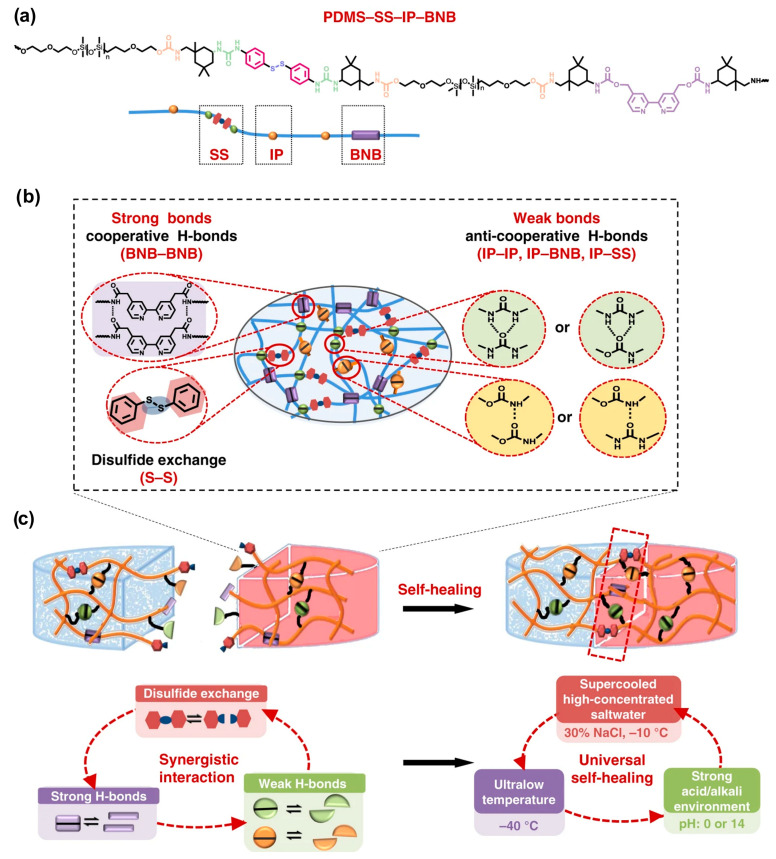
Chemical structure (**a**) and self-healing mechanisms in PDMS–SS–IP–BNB copolymers (**b**,**c**) [61] Creative Commons CC BY license.

The synergistic interaction of multiple dynamic bonds including disulfide metathesis and a combination of strong and weak H-bonds was also exploited for the preparation of a highly stretchable PDMS-based self-healing elastomer both unnotched and notched samples, designed for applications in harsh conditions [61]. The materials were prepared with telechelic dihydroxyethylpropoxyl-PDMS (*M_w_* = 4600 g mol^−1^) and isophorone diisocyanate (IPDI) to form bis-isocyanate-terminated preoligomer. Chain extenders (4,4′-dithiodianiline (SS) and/or 4,4′-bis(hydroxymethyl)-2,2′-bipyridine (BNB)) were then added at varying ratios to obtain a hybrid PDMS–SS–IP–BNB copolymer (Scheme 20). Non-covalent and covalent inter- and intra-chain interactions were synergistically involved in the improvement of their mechanical strength.

Rheological tests under ambient conditions showed mostly the elastic deformation of PDMS–SS–IP–BNB, whereas at T > 60 °C an enhanced mobility of macromolecules was noted and a solid–liquid transition to viscous deformation. The mechanical properties of the material depend on the ratio of the PDMS, SS, BNB and IP units. The stress–strain curves included an initial stiffening region, followed by a stable region until fracture. The presence of flexible and soft PDMS segments was important for the materials elasticity. The stretchability of PDMS–SS–IP–BNB was highly affected by the strong quadruple H-bonding between PNB-PNB and depended on the loading rate. Weaker H-bonding that involved IP units (IP–IP, IP–BNB or IP–SS interactions) was important for efficient strain energy dissipation by the reversible rupture of sacrificial bonds. The dynamic metathesis of aromatic disulfides also influenced the tensile strength.

The presence of SS and BNB units did not disrupt the PDMS-based soft domain, thus enabling it to self-heal in universal conditions. Autonomous self-healing occurred within a wide temperature range owing to a low *T*_g_ of polysiloxane backbone (self-healing efficiency, *η*_s_~50% after 10 min at room temperature and 93% after 2 h; 52% after 24 h at −40 °C); underwater (93% healing efficiency after 24 h due to the hydrophobicity of PDMS segments); in 30% NaCl solution at −10 °C (89% efficiency after 24 h); and in a strong acid/alkali environment (pH = 0 or 14, 88% or 84% efficiency after 24 h).

The crosslinked elastomer was also integrated as the encapsulator and protecting coating with a eutectic EGaIn alloy, thus demonstrating the material application toward a stretchable self-healing artificial conductor. The electrical conductivity was maintained despite being stretched to 400% strain or cut and then reconnected (immediate restoration of electrical conductivity and recovery of mechanical properties after 10′). The universal autonomous self-repair features and good mechanical properties make PDMS–SS–IP–BNB suitable for applications in self-healing electronic equipment in marine environments, polar regions or industrial wastewater, artificial e-skin applications and durable electronic communication devices operating under harsh conditions.

**Scheme 21:**
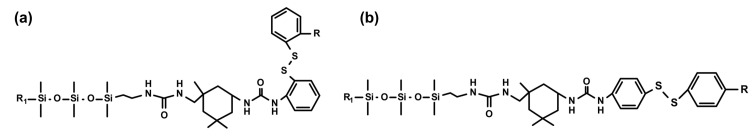
Chemical structure of polysiloxanes terminated with disulfides of (**a**) ortho- and (**b**) para-position on aromatic rings.

A combination of dynamic disulfide bonds and hydrogen-bonded urea segments was also applied for self-healing of a siloxane-based hybrid material, designed as a passive de-icing coating of extended service life [62]. Hydrogen bonds of the urea groups and Van der Walls interactions between benzene rings participated in this process of self-organization and phase separation in the silicones. The asymmetric position of the sulfur atoms on the benzene ring (position ortho) (Scheme 21) resulted in a more loose packing of hard segments in those materials which facilitated the migration of these structures to the surface and contributed to their improved self-healing efficiency (*η*_s_~99%).

The higher content and regular disulfide moieties (position para) enhanced the crystallization of urea motifs. As a consequence, more silicone segments were exposed on the surface, which contributed to the enhancement of hydrophobic properties. Along with a low Young’s modulus, it was beneficial for low ice adhesion force (~7 kPa). The ice repelling behavior was maintained after healing.

Self-healing silicone elastomer crosslinked with hydrogen bonds and dynamic disulfide linkages may be combined with interactions of different types. An example can be a silicone elastomer functionalized with thioctic acid, grafted at terminal positions of PDMS chains by coupling with aminopropyl groups that was modified with 2,6-pyridine dialdehyde [63] that was designed as a coating for soft robotics or wearable/stretchable electronics. Pyridine-(bis)imine moieties acted as Fe^3+^-binding ligands whereas 1,3-diisopropenylbenzene (DIB) was used to stabilize the polysiloxane network. The resulting self-healable elastomer was thermally stable, stretchable, exhibited strong adhesion affinity and anticorrosion performance. 

#### 3.1.3. Silicone Elastomers Regeneration by Synergistic Action of H-Bonding between Urea Groups and Dynamic Imine Bonds

**Scheme 22:**
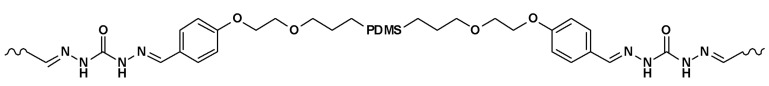
Synthesis of dual-crosslinked polysiloxanes with bis-acylhydrazone units.

The mechanism of self-healing in silicone elastomers may involve the synergistic combination of hydrogen bonds and dynamic covalent Schiff-based imine bonds. Roy et al. obtained silicone networks that were simultaneously double dynamic due to reversible covalent imine bonds and dynamic non-covalent hydrogen-bonded urea groups within the same supramolecular silicone framework [64]. A soft stretchable dynamic polysiloxane with bis-iminourea-type (bis-acylhydrazone) subunits was obtained by polycondensation of bis-tosyl aldehyde functionalized siloxane oligomers and carbohydrazide monomeric components (Scheme 22).

**Figure 8:**
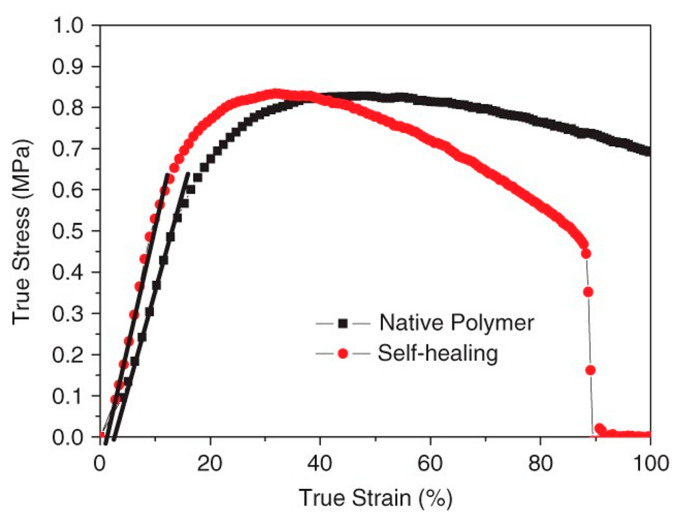
Stress–strain curves of the native and self-healed film of polysiloxanes with bis-acylhydrazone units. Reprinted from [64], Copyright (2014), with permission from John Wiley & Sons, Inc.

The dynamic polymer underwent self-healing in the course of a few hours at room temperature, after bringing together and gentle pressing of the cut surfaces for a few minutes, without the application of additional pressure or load. Shorter healing (<4 h) was not efficient. It was shown that regeneration under such conditions predominantly involved hydrogen bonding. The mechanical properties were recovered with *η*_s_~90%. The stress–strain curves (Figure 8) displayed nearly identical elastic regions of the native and self-repaired polymers, indicating the restoration of the molecular network upon healing. However, the native polymer had a slightly higher ultimate stress and it underwent permanent deformation at >115% strain. The self-healed polymer’s rupture stress was ~0.5 MPa and the sample underwent permanent deformation upon true strain >90%. 

**Scheme 23:**
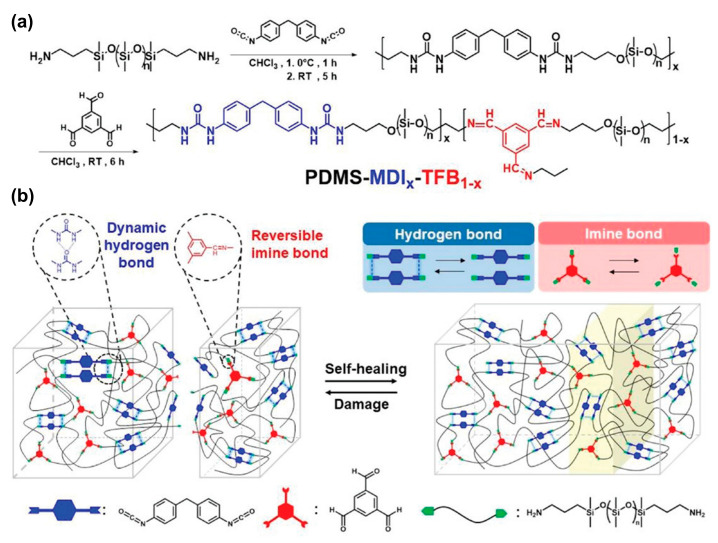
Synthesis of PDMS-MDI*_x_*-TFB_1−*x*_ (**a**) and mechanism of its self-healing based on the synergistic effect of reversible weaker imine bonds and stronger hydrogen bonds (**b**) [65]. Creative Commons CC BY license.

More recently, supramolecular water-assisted self-healing polymers PDMS-MDI_x_-TFB_1−x_ were described [65]. They displayed an excellent self-healing kinetic behavior and high stretchability due to the reversible dissociation–association of hydrogen and imine bonds. The elastomers were prepared with telechelic aminopropyl terminated PDMS oligomers as hydrophobic flexible blocks, methylene diphenyl diisocyanate (MDI) as a precursor of hard urea-based segments that formed strong intermolecular H-bonds to crosslink the network and triformaldedhyde benzene (TFB) units to generate weak, covalent, reversible imine bonds as Schiff-base reaction products (Scheme 23). This composition guaranteed a hydrophobicity/hydrophilicity balance in the network.

**Figure 9:**
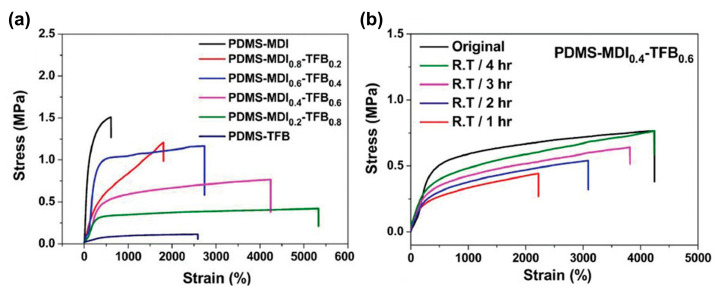
Mechanical properties of PDMS-MDI*_x_*-TFB_1−*x*_ (**a**) and progress of S-H with time (**b**) (stress–strain curves at loading rate of 20 mm min^−1^) [65]. Creative Commons CC BY license.

The mechanical performance of PDMS-MDI_x_-TFB_1−x_ at room temperature was very good (tensile strain of ~4200%, and toughness of 26.8 MJ m^−3^) and their self-healing was effective (*η*_s_ of 90% after 4 h) (Figure 9). The elastomers’ mechanical properties were maintained due to H-bonds between urea segments whereas the imine metathesis enabled the efficient diffusion of strain energy along cracks.

**Figure 10:**
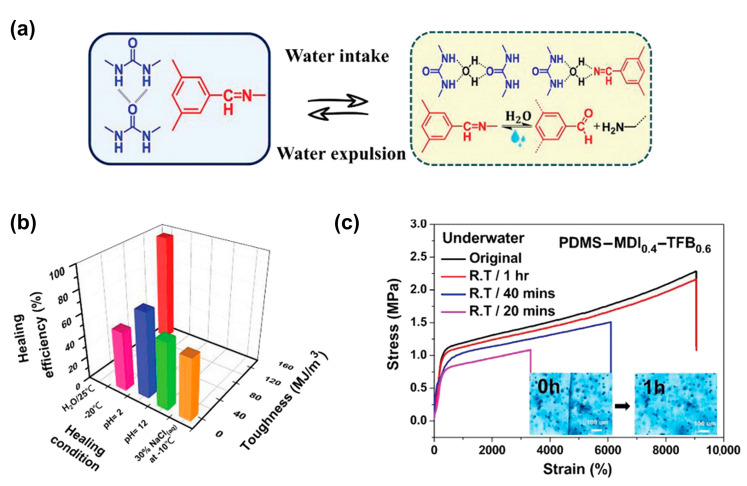
(**a**) Schematic diagrams of water-assisted self-healing mechanism involving urea and imine bonds, (**b**) efficiency of self-repair of PDMS-MDI0.4-TFB0.6 in different harsh conditions and self-healing capability and (**c**) stress–strain curves of PDMS-MDI_0.4_-TFB_0.6_ self-healed underwater at room temperature for different healing times (inset: optical microscope image (6 × 4 mm) of the damaged film and after 1h of healing) [65]. Creative Commons CC BY license.

Water molecules facilitated the reversible process of association/dissociation of crosslinks and influenced the self-healing kinetics of the network rearrangements that involved H-bonds and imine bonds (Figure 10). The hydrophobic effect and polymer matrix plasticization on the action of H_2_O molecules were attributed as the driving force of the observed phenomena. Most importantly, water-assisted self-healing polymers displayed improved characteristics after curing (tensile strain of 9050%, self-healing efficiency of 95% within 1 h and toughness of 144.2 MJ m^−3^) (Figure 10). The effect was tentatively ascribed to the entropic penalty originating both from PDMS conformational entropy decrease and water-induced shifting of energetically unfavorable van der Waals interactions that would accelerate the polymer recovery.

Moreover, a WASHP-based light-emitting touch-responsive device (WASHP-LETD) of high adhesiveness and a favorable mechanical deformation performance under pressure, bending, and strain; as well as perovskite quantum dot (PeQD)-based white LED backlight (long-term stability in a boiling water environment) were prepared as waterproof components to be integrated into next-generation e-skin devices, wearable electronics and flexible displays.

**Scheme 24:**
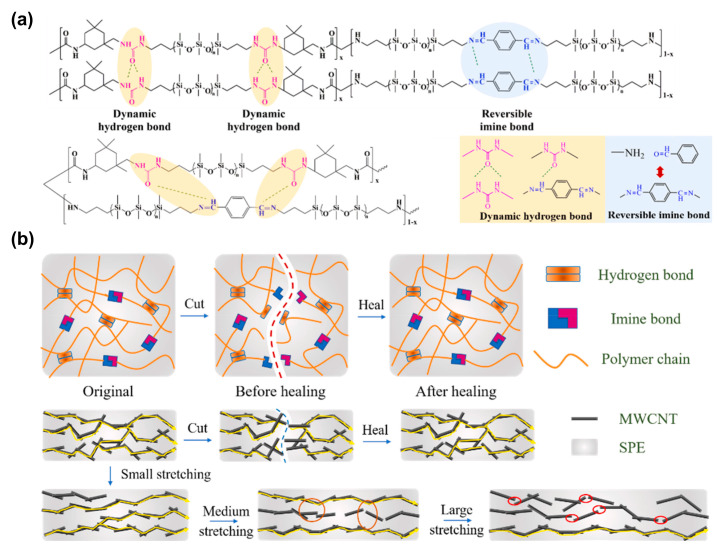
(**a**) Synergistic interactions formed between multiple dynamic bonds and (**b**) schematic illustration of self-healing and sensing mechanisms operating in ESFSS. Reprinted from [66], Copyright (2023), with permission from Elsevier.

A similar synergistic action of intermolecular dynamic hydrogen bonding between urea groups and reversible metathesis of imine bonds was used in a PDMS-based self-healable elastomer blended with multi-walled carbon nanotubes (MWCNT) [66]. The composite was employed in a high electrical self-healing flexible strain sensor (ESFSS) for human motion detection. The hybrid elastomer was obtained in *co*-polycondensation of H_2_N-(CH_2_)_3_-PDMS-(CH_2_)_3_-NH_2_ and isophorone diisocyanate (IPDI) and then terephthalaldehyde (TA) resulting, respectively, in the formation of urea and imine groups in the main polymer chain (Scheme 24). Strong intermolecular crosslinks were generated by hydrogen bonds between urea–urea and urea–imine groups, whereas dynamic imine bonds acted as reversible crosslinking points. A complete healing of this material (*η*_s_ = 98.3%) occurred after 18 h at 60 °C (or *η*_s_ = 95.4% at 35 °C for 48 h). MWCNT formed a uniform conductive network in the crosslinked elastomer and the conductive pathway was rebuilt when the nanotubes were pulled to contact each other during the re-mending of the elastomer matrix (Scheme 24).

**Figure 11:**
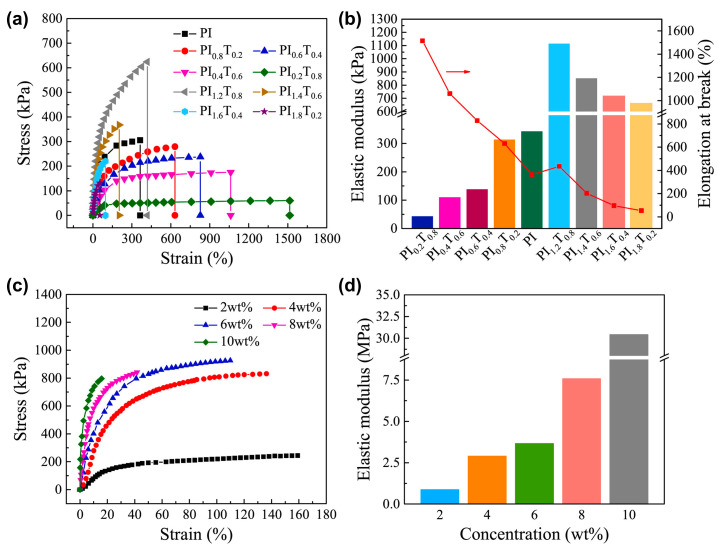
(**a**) Stress–strain curves and (**b**) elastic modulus and elongation at break for various SPE composition. (**c**) Stress–strain curves and (**d**) elastic modulus of the ESFSS prepared with different MWCNT contents. Reprinted from [66], Copyright (2023), with permission from Elsevier.

The characteristics of flexible strain sensors, including the electrical self-healing performance along with mechanical properties, could be adjusted by changing the initial material ratio of the components (Figure 11).

The elongation at the break of the SPE decreased from 1515% to ~54% with the increase in the urea content. Young’s modulus (44.31 kPa–1116.05 kPa) first increased and then decreased with the increase in the amount of urea bonds. The best mechanical performance parameters (632% elongation at break, 269.95 kPa tensile stress and 314.89 kPa Young’s modulus) are comparable to those of the epidermis layer of human skin and can meet the requirements for strain sensors.

The best overall performance (mechanical, electrical properties and self-healing behavior) was displayed at 4 wt.% carbon nanotubes content. The elongation at break decreased while the tensile strength increased with the increase in MWCNT content. However, the composites’ electrical healing efficiency decreased with the increase of MWCNT content (no self-heal at 10 wt.% of MWCNT despite high conductivity). The healed ESFSS presented the same sensing performance as the original sample. A successful detection of motion at different parts of the human body was demonstrated in real time as a response to the applied strain.

**Scheme 25:**
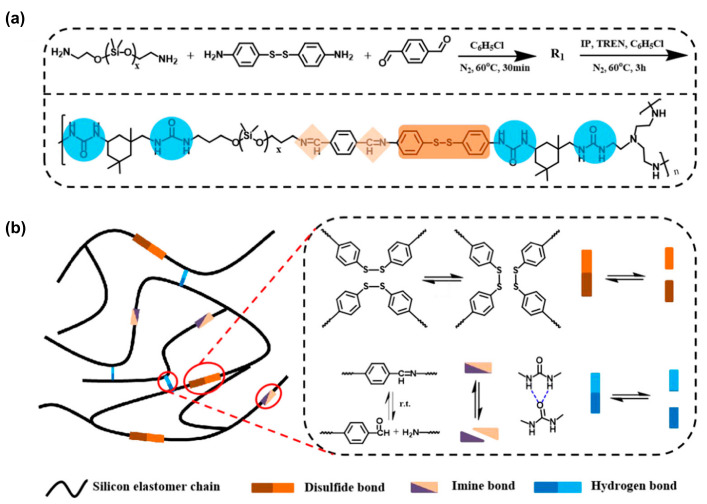
Synthesis and molecular chain structure of ternary silicone elastomers (**a**) and schematic diagram of the operating self-healing interactions (**b**). Reprinted with permission from [67]. Copyright (2021) American Chemical Society.

The introduction of imine bonds to stretchable silicone elastomers, crosslinked by means of dynamic disulfide bonds and strong H-bonding (Scheme 25), not only improved their S-H effectiveness at ambient conditions and in harsh environments, but also enhanced their adhesion to metals (copper, iron and aluminum) and nonmetals (paper, PI, PLA, PP and glass) [67].

**Figure 12:**
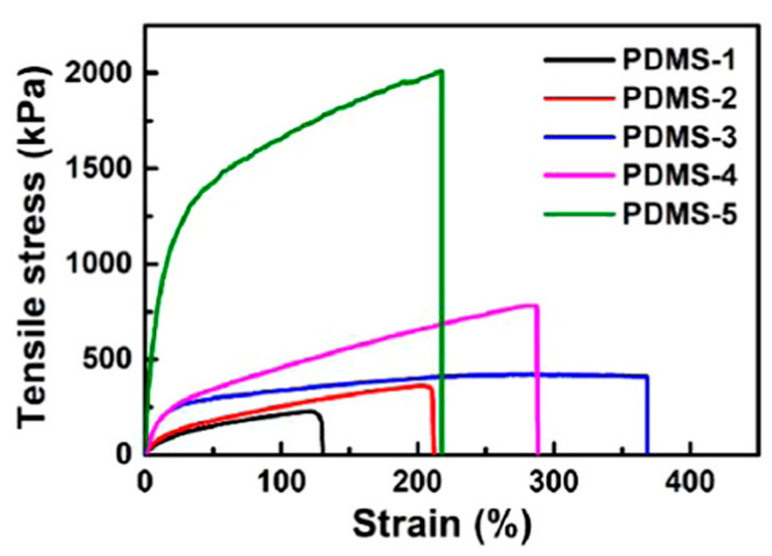
Tensile stress–strain curves of PDMS-1-5 (respective molar ratios in PDMS-1-5: H_2_N-PDMS-NH_2_/APD/TREN = 3:1:0.5; IP: 2, 2.5, 3, 3.5, 4.5 and TPA: 2.5, 2, 1.5, 1, 0). Adapted with permission from [67]. Copyright (2021) American Chemical Society.

Mechanical properties of the ternary elastomers could be tuned by changing the content of hydrogen and imine bonds (Figure 12). The synergistic effect of multiple dynamic bonds in a hard segment resulted in a good stretchability (ε_max_ = 368%, *σ*_max_ = 1.9 MPa). The maximum stress gradually increased on increasing the amount of hydrogen bonds, while the elongation at break first increased but then decreased. Furthermore, energy dissipation due to the reversible association of dynamic bonds was shown.

The self-healing properties were displayed (*η*_s_ of 98.1%, after healing PDMS-3 for 5 h at room temperature, and 96.4% after 5 h in aqueous environment) along with stable adhesion in acidic (pH 1) and alkaline (pH 12) environments, salt water, petroleum ether, water, etc. The re-mending efficiency depended on the structure of the elastomer, especially the content of disulfide bonds. The role of crosslinking by disulfide bonds and entanglements enhancement was highlighted by the effect of replacement of 4,4′-diamino diphenyl disulfide ether (APD; polymer PDMS-2) with 4,4′-diaminodiphenyl methane unit (polymer PDMS-0). It resulted in a significant improvement of the tensile properties (from 90.4 to 219.0%). The S-H effect was enhanced by the intrinsic hydrophobicity of the ternary silicone elastomers that in addition could be reversibly deformed by stretching, twisting and knotting. Such materials can potentially be used as fracture- and rust-resistant protective coatings or sealings, as well as for manufacturing smart e-skin and in soft robotics.

#### 3.1.4. Silicones S-H by Synergistic Effect of H-Bonding and Other Supramolecular Interactions

**Scheme 26:**
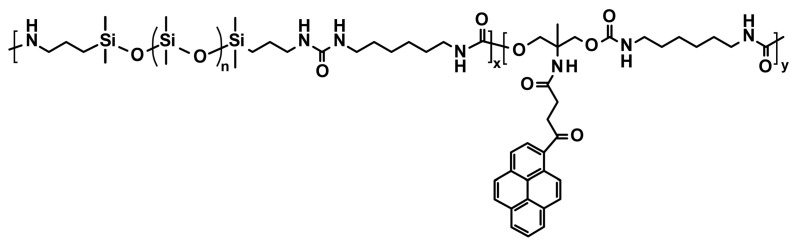
Structure of H-PDMS-Py*_m_*.

Supramolecular silicones often incorporate other structural segments capable of supramolecular interactions, including *π*–*π* stacking and host–guest interactions. A recyclable siloxane elastomer (H-PDMS-Py*_m_*) (Scheme 26), self-healing through a system of combined strong and weak hierarchical hydrogen bonds between urea groups and urethane groups, incorporated additionally pyrene chromophores [68]. The strong π–π interactions due to Py groups stacking enhanced microphase separation in H-PDMS-Py*_m_*.

H-PDMS-Py*_m_* was highly stretchable and its mechanical and self-healing properties could be tuned by varying the ratio of *π*–*π* stacking to hydrogen bonding. Both inter- and intra-chain H-bonds between urea and urethane groups took part in energy dissipation and enhancement of the elastomer’s toughness (high tensile strength *σ*_max_ of 7.46 MPa and strain at break ε_max_ of 2174%). The impact of the hydrogen bonding depends on the spatial proximity of the interacting groups. With the increase of pyrene groups’ content, the tensile strength increased because of the stabilizing *π*–*π* interactions. The energy dissipation efficiency was reduced since most of the broken sacrificial bonds could be reformed only after a longer relaxation time. Such materials may be potentially used in stretchable electronics and flexible sensors. The supramolecular phenomena were also responsible for a high self-healing rate of H-PDMS-Py*_m_* (*η*_s_ = 96% after 1 h at 110 °C). The healing efficiency was also related to the content of Py groups (it gradually declined when the amount of pyrene groups increased). Interestingly, H-bonding and π–π stacking interactions in this system showed different trends in their thermal reversibility. Disassociation–association of H-bonding occurred at 80 °C. The acceleration of the healing rate by increasing temperature can also be linked to improved diffusion of polymer chains.

The π–π interactions were also applied in a self-healing PDMS obtained by the incorporation of a cyclometalated platinum(II) 6-phenyl-2,2′-bipyridyl complex into the polysiloxane backbone [69]. The synergistic combination of dynamic Pt···Pt and π–π molecular interactions was strong enough to crosslink the linear polysiloxane chains into an elastic film. When damaged, the polymer can be healed at room temperature without any healants or external stimuli even after the surface ageing for 12 h due to the hydrolytic inertness of Pt(II) complexes. The tensile modulus and modulus of toughness were recovered after 12 h under those conditions (and within 2 h at 50 °C or 1 h at 80 °C). High stretchability of the supramolecular film was noted (over 20 times of the original sample length) since the combination of Pt···Pt interactions and π–π interactions was strong enough to sustain stretching. 

**Scheme 27:**
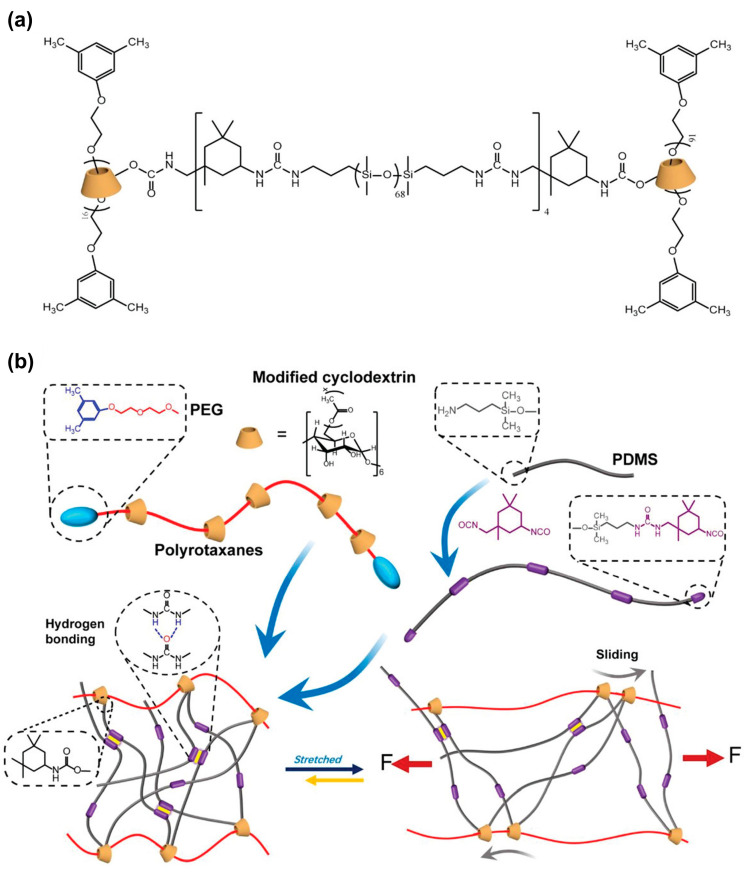
Molecular structure of polyrotaxanes-embedded PDMS (**a**) and schematic presentation of the dual crosslinking and the behavior of siding polyrotaxanes (**b**). Reprinted from [71], Copyright (2020), with permission from John Wiley & Sons, Inc.

Cyclodextrin-based host–guest non-covalent interactions display a reversible nature and can be also exploited in S-H systems [70]. Incorporation of modified cyclodextrins as sliding crosslinkers on polysiloxanes containing urea motifs resulted in a highly stretchable and self-healable elastomer (Scheme 27) with good mechanical strength [71]. The elastomer of optimized composition displayed good mechanical performance (ε_max_ 2800% with a fracture strength of 1.05 MPa; fracture toughness of 6765 J m^−2^) and self-healed nearly completely (*η*_s_ = 93%) at 55 °C. The mechanical properties of this system were attributed to the combined effects of sliding cyclodextrins along guest chains and hydrogen bonds in the dual crosslinking system. When the polymer was highly stretched, hydrogen bonds would break and the polyrotaxane system would slide to additionally dissipate energy. The crosslinking density increased as the amount of cyclodextrins decreased, which was reflected in a corresponding increase of storage modulus (*G*′) of the samples (G′ > loss modulus G″ for all the studied materials). The sample of lowest density of H-bonded urea crosslinks was most stretchable (ε_max_~3000%) but had low fracture strength (~0.3 MPa). On the other hand, the sample with a low amount of polyrotaxanes exhibited a high stress at break (1.2 MPa) but its stretchability was reduced to ~2500%. 

The elastomer was used to fabricate a strain sensor by coating single-walled carbon nanotubes (SWCNTs) on the surface of the hybrid stretchable elastomer (by dipping SWCNTs solution onto the substrate). The resulting sample was used to effectively detect human movement and could potentially be used in the fabrication of stretchable electronic devices.

Host–guest interactions involving β-CD were also applied in other PDMS-based systems. The addition of poly(3,4-ethylenedioxythiophene/permethylated β-cyclodextrin) polypseudorotaxane (PEDOT-PMβCD) to PDMS resulted in a decrease of Young’s modulus (<0.2 MPa), enhanced elongation at break (<735%) and dielectric permittivity (~3.5), despite a weak phase separation behavior [72]. The hybrid material was applied in an actuator that displayed good electromechanical performance. PDMS was also modified in order to obtain a macroscopic supramolecular assembly (MSA) based on the host/guest molecular recognition between the supramolecular surface groups of β-cyclodextrin and adamantane prepared via a layer-by-layer (LbL) assembly technique [73]. The effect of interactive forces between the modified PDMS building blocks of different elastic moduli (0.38 to 3.84 MPa) influenced the observed varied elastic modulus range. The tests of MSA of PDMS by the formation of assembled dimers in water proved that the effect gradually declined from 100% at 0.38 MPa to 0% at 3.84 MPa.

### 3.2. Self-Healing of Polysiloxanes Based on H-Bonding and Metal Cations’ Coordination

Good self-healing ability and high stretchability, typically achieved in hydrogen-bonded siloxanes, enables the rapid reformation of the elastomers after the fracture and dissipation of strain energy on stress. Importantly, organic groups containing heteroatoms may also be applied as ligands for the coordination of metal cations. In this way, they can act as secondary crosslinking points in H-bonded siloxane elastomers.

**Scheme 28:**
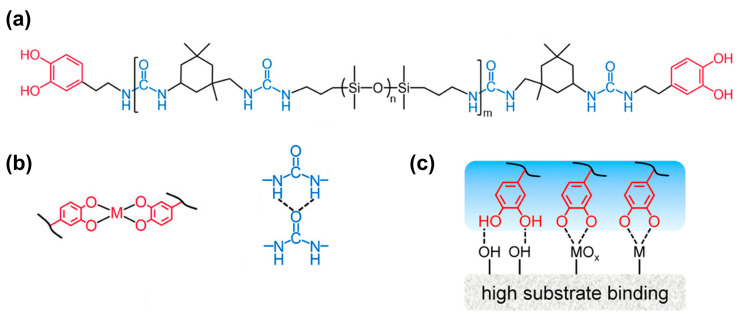
Chemical structure of PDMS-CatN (**a**), the structure of dual-crosslinks in supramolecular network (**b**) and interactions of catechol groups with surface (**c**). Adapted with permission from [74]. Copyright (2019) American Chemical Society.

The supramolecular design of dual-crosslinked polysiloxanes (PDMS-CatN) of this type was applied to thermally healed polymer coatings with ion-controlled mechanical properties and coloration [74]. The silicone networks were formed by interactions of hydrogen bonding of urea derivatives and metal–catechol complexes (Scheme 28).

The density of the supramolecular crosslinks in PDMS-CatN was regulated by tuning either the feed ratio of monomers (PDMS, IPDI and DOPA) or the chain length of the poly(dimethylsiloxane) segments. The type of metal ions influenced the color and mechanical properties of the networks because of various metal–ligand binding abilities (Table 1). The elastomers were phase-separated but formed highly adhesive coatings on various substrates that were recoverable from mechanical damage and showed oil repellency.

**Scheme 29:**
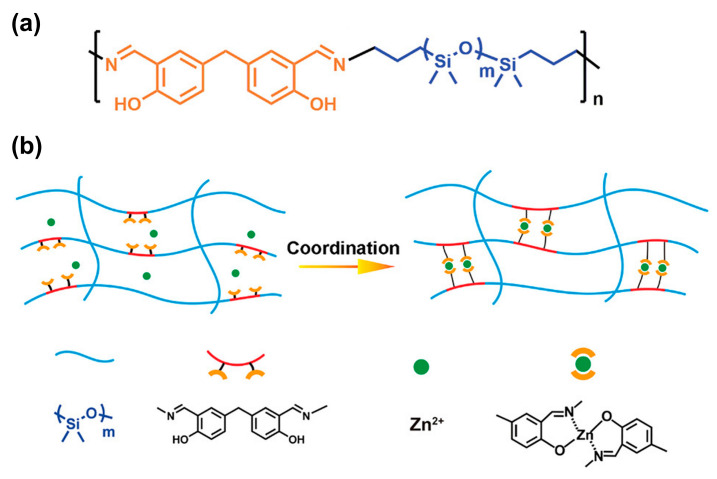
Chemical structure of PDMS-salicyaldimine copolymers (**a**) and preparation of supramolecular networks by coordination of Zn^2+^ ions (**b**). Adapted from [75], Copyright (2021), with permission from John Wiley & Sons, Inc.

Mechanically strong, self-healing and recyclable, photoluminescent silicone elastomers were prepared by coordination bonding of Zn^2+^ ions to salicylaldimine motifs embedded in PDMS (Scheme 29) [75]. The synergistic coordination of N and O atoms toward Zn^2+^ ions resulted in good mechanical performance. The supramolecular transparent elastomers exhibited a tensile strength up to 10 MPa with strain-at-break ε_max_ of ~226% (sample P1-Zn-0.5), good thermal stability and coordination-enhanced fluorescence.

**Figure 13:**
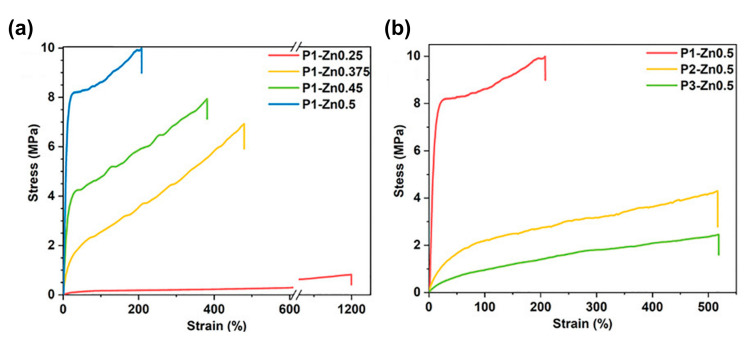
Stress–strain curves of elastomers (**a**) with varying Zn/imine molar ratios and (**b**) with different chain lengths of PDMS precursors (P1: 1000 g mol^−1^; P2: 2000 g mol^−1^; P3: 3000 g mol^−1^). Adapted from [75], Copyright (2021), with permission from John Wiley & Sons, Inc.

The mechanical properties and stress relaxation kinetics were tuned by adjusting the length of PDMS segments or the content of metal ions (Figure 13). With the decrease of the metal–ligand ratio (from 0.5 to 0.25) and the crosslinking density, the tensile strength of the material decreases (from 10.0 to 0.825 MPa), while the elongation at break increases (from 226% to 1200%). The length of the PDMS block affects the crosslinking density without changing the kinetics of ligand exchange.

**Figure 14:**
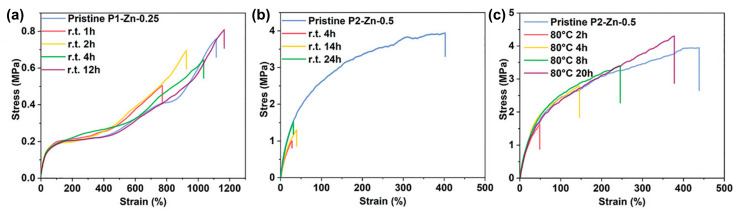
Tensile curves of (**a**) P1-Zn-0.25 and (**b**) P2-Zn-0.5 healed at room temperature and (**c**) P2-Zn-0.5 healed at 80 °C for different times. Adapted from [75], Copyright (2021), with permission from John Wiley & Sons, Inc.

The elastomers self-healed under mild conditions due to the rapid exchange of Zn-salicyaldimine interactions and could be reprocessed via hot pressing or chemical cycling due to the dynamic nature of metal coordination. The more dynamic networks (lower amount of coordination crosslinks) showed higher self-healing efficiency (Figure 14). P1-Zn-0.25 recovered completely within 1 h at room temperature, and even showed 109% of its original toughness after 12 h. In contrast, only 38.8% of the original tensile strength and 7.79% of the original strain was recovered in P2-Zn-0.5 after 24 h.

At elevated temperatures, the mobility of polymer chains and the rate of ligand exchange can be increased, leading to accelerated diffusion of macromolecules across the cut interface and faster dissociation/association equilibrium of the dynamic crosslinks. About 85% recovery of the pristine P2-Zn-0.5 sample’s toughness was noted after 20 h at 80 °C.

The elastomers have also a potential as photoluminescent elastomers or coatings. In solution, the intensity of characteristic emission peaks at 433 nm (*λ*_ex_ = 274 nm) was significantly enhanced upon coordination with zinc ions. This chelation-enhanced fluorescence emission was attributed to the suppressed C=N isomerization and excited-state intramolecular proton transfer by coordinating with N and O atoms. In the solid-state, luminescence could be excited by a broad range of UV (λ = 270–470 nm). Bright green fluorescence (510 nm) was emitted by P1-Zn-0.5 film under irradiation at 365 nm.

A similar synergistic coordination of N and O atoms towards various metal ions (Fe^3+^, Zn^2+^ and Al^3+^) featured in coordinative polysiloxanes functionalized with side 2-hydroxy-1-naphthyl imine groups [76]. A remarkable ability to recognize metal ions was demonstrated by dynamic elastomers featuring reversible coordination and imine bonds. The interaction of metal ions with the organic ligands in these silicones yielded thermoplastic S-H polymers that can be exploited as reprocessable shape memory materials due to the combined effect of glass transition and topological rearrangement. Elastomer P1-10%-Fe^3+^ exhibited high mechanical strength (*σ*_max_ 8.8 MPa) and fracture toughness (33.22 MJ/m^3^).

**Scheme 30:**
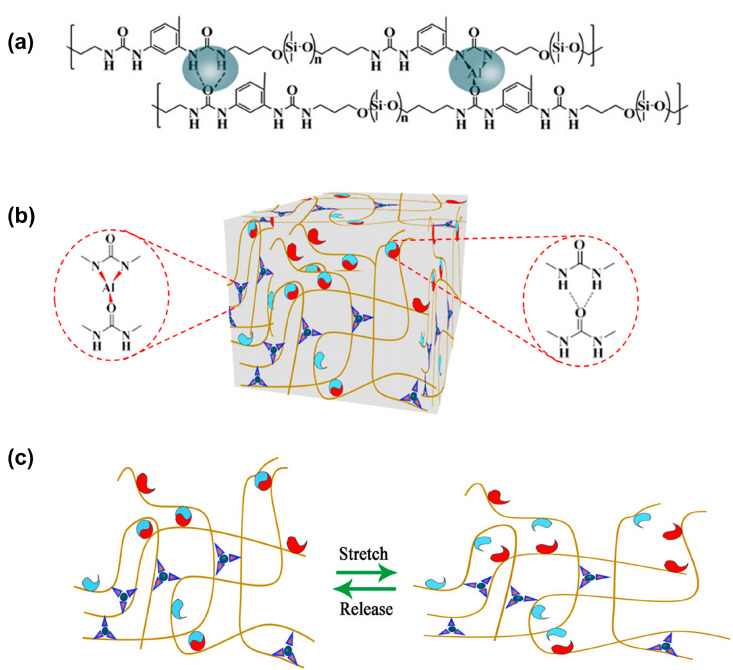
Molecular structure of PDMS-TDI (**a**), schematic presentation of the dual crosslinking in Al(III)-coordinated PDMS–TDI (**b**), and the postulated mechanism of its behavior upon stretching (**c**). Adapted with permission from [77]. Copyright (2019) American Chemical Society.

The effects of coordination of metal ions to quadruple hydrogen bonds between urea groups can be desirable. A self-healing supramolecular polysiloxane (PDMS–TDI) of this type, with urea units in the main chain, was developed via a one-pot polycondensation of bis(3-aminopropyl)-terminated poly(dimethylsiloxane) and 2,4′-tolylene diisocyanate (TDI), followed by the coordination of urea segments with Al(III) ions (Scheme 30) [77].

**Figure 15:**
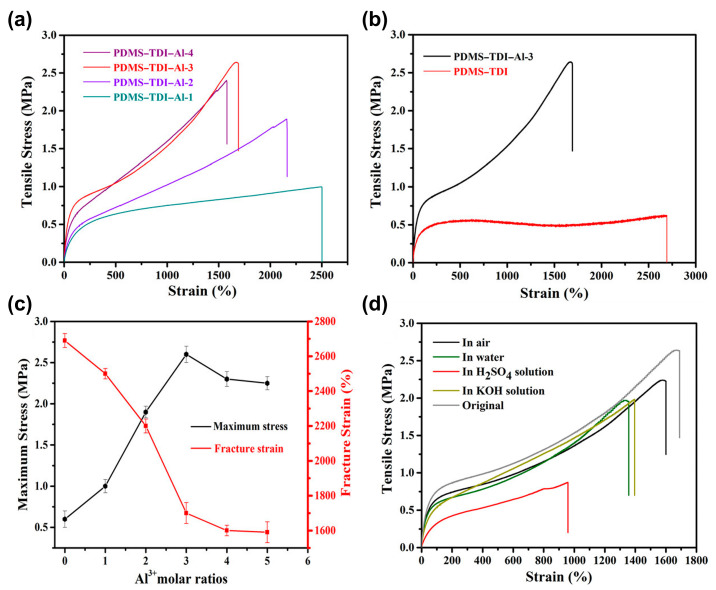
Stress–strain curves ((**a**) deformation rate of 20 mm min^−1^) representing mechanical properties of PDMS–TDI and PDMS–TDI–Al (**a**), series of PDMS–TDI–Al with different content of Al(III) (**b**), fracture strain and maximum stress dependence of PDMS–TDI–Al on molar ratios of Al(III) (**c**) and self-healing of PDMS–TDI–Al under different conditions (**d**). Adapted with permission from [77]. Copyright (2019) American Chemical Society.

A robust molecular network was prepared by the formation of strong dynamic coordination bonds to urea groups. It improved mechanical properties of the hybrid polymer (*σ*_max_ up to ~2.6 MPa, toughness~14.7 MJ/m^3^, ε_max_~1700% for PDMS-TDI-Al-3 (Figure 15a–c)), and provided an excellent self-healing ability (*η*_s_ = 90%). The polymer was also healable under harsh conditions (Figure 15d). These parameters indicate a potential for the fabrication of flexible electronic skin.

**Scheme 31:**
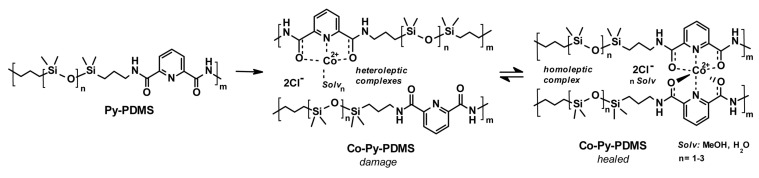
S–H mechanism based on formation of Co-Py-PDMS.

**Scheme 32:**
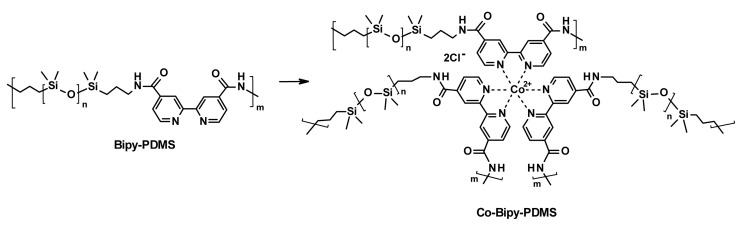
S–H mechanism based on formation of Co-Bipy-PDMS.

The structure of the ligands and the type of chelation can be important for the properties of elastomers. The structural features of tri- or bidentate polymer–metal complexes cobalt(II)pyridinedicarboxamide-*co*-polydimethylsiloxane (Co-Py-PDMS) and cobalt(II)-bipyridinedicarboxamide-*co*-polydimethylsiloxane (Co-Bipy-PDMS) (Co^2+^ content 0.09–2.41 wt.%) provided high chelation ability in 3D networks [78]. Their mechanical properties were controlled by the coordination binding of *O*,*N*,*O*- (Scheme 31) and *N*,*N*-chelating (Scheme 32) as well as by the length of the polysiloxane unit (*M*_n_: ~900, 5000, or 25,000 g·mol^−1^). The crosslinked networks exhibited shelf stability in air and good resistance to humidity, which is promising for such applications as highly elastic S-H membranes or coatings for, e.g., optoelectronic devices.

**Figure 16:**
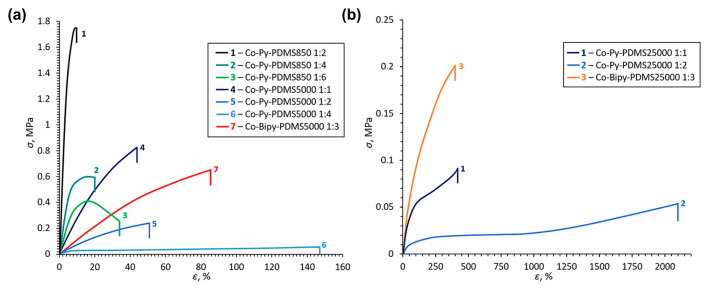
Stress–strain curves of (**a**) Co-Py-PDMS850s, Co-Py-PDMS5000s and Co-Bipy-PDMS5000, (**b**) Co-Py-PDMS25000s and Co-Bipy-PDMS25000, (stretching rate 40 mm·min^−1^). Adapted with permission from [78]. Copyright (2021) American Chemical Society.

The multidentate chelation resulted in a high Young’s modulus (up to 35 MPa), tensile strength (up to 1.75 Mpa) and elongation at break (up to 2100%), depending on Co^2+^ content and the structure of the ligand (Figure 16). Changing the main-chain ligand from Py-PDMSs to Bipy-PDMSs led to a (2–4)-fold increase in tensile strength. However, room temperature self-healing efficiency was ~96% for Co-Py-PDMSs and ~40% for Co-Bipy-PDMSs (PDMS *M*_n_ = 25,000 g·mol^−1^). The length of the polysiloxane segment also affected the network dynamics (for Co-Py-PDMS25000 1:2 and Co-Py-PDMS5000 1:4, the self-healing efficiencies of 80 and 90% were achieved after 24 h).

A dual-crosslinked PDMS-based polymer network incorporating two potential ligands of Fe^3+^ ions was prepared by the synergistic combination of strong and weak metal–ligand interactions [79]. The polymer (NAC-pdca-PDMS) was obtained by the co-condensation of 3-aminopropyl end groups of poly(dimethyl-*co*-methylvinyl)siloxane with 2,6-pyridinedicarboxamide (pdca), and the addition of N-acetyl-l-cysteine to the side chain vinyl groups. NAC-pdca-PDMS was subsequently treated with ferric ions.

The co-existence of stronger Fe/N-acetyl-l-cysteine (Fe/NAC) and weaker Fe/pyridinedicarboxamide (Fe/pdca) coordination sites governed the mechanical properties of the network. Fe/pdca served as sacrificial bonds to dissipate energy through bonds breaking, exchanging or reforming whereas Fe/NAC played the role of permanent bonds and helped to retain the network integrity and elasticity.

The elastomer exhibited good mechanical strength and toughness as well as moderate viscoelasticity, while showing autonomous self-healing ability at room temperature. These properties could be tuned by varying in the ratio of Fe/pdca to Fe/NAC through the addition of different amount of Fe^3+^ ions. The introduction of larger amounts of Fe^3+^ or increasing the healing temperature enhanced coordination, and resulted in stronger mechanical properties of the reprocessed samples.

Fe/pdca interactions were not beneficial for the tensile strength and were responsible for a relatively high elongation at break, but low tensile strength (respectively, ε_max_~547% and *σ*_max_~0.08 MPa). In contrast, sample Fe/NAC-pdca-PDMS 0.5:1 exhibited both increased deformation (ε_max_ 627%) and tensile strength (*σ*_max_ 0.21 MPa) (elongation at break in a control sample Fe/control-2 was ~162% and *σ*_max_~0.20 MPa). However, the healing rate decreased with the increasing amount of Fe/NAC coordination sites. Thin films of Fe/NAC-pdca-PDMS 0.5:1 and Fe/NAC-pdca-PDMS 1:1 sustained a large strain after breaking for 8 h and after 16 h (the healing was >90% of the original value). The effect was attributed to shifting ferric ions from Fe/pdca to Fe/NAC or the internal transformation of Fe/NAC complexes. The latter option can be supported by the heating–hardening bbehavior that was also observed in the reprocessed Fe/NAC.

PDMS-based oligomers with N-ligands were studied with regard to their self-healing efficiency through the variation of the nature of the metal–ligand interactions used [80]. The materials were prepared through *co*-condensation between pyridine-2-carboxaldehyde and aminopropyl-terminated PDMS (1000 Da) to generate N-ligands from pyridine and imine functional end groups. Co(BF_4_)_2_, Fe(BF_4_)_2_ and Zn(BF_4_)_2_ metal salts were used to study the metal–ligand coordination effect (geometry of the complex and bond strengths) on the thermomechanical properties of the system, whereas the influence of counter-ion was probed by utilizing Zn(ClO_4_)_2_, Zn(OTf)_2_ and Zn(BF_4_)_2_ salts. Independently of the metal ion source, octahedral metal complexes were generated that crosslinked the supramolecular network.

Various factors, including size, coordinating ability and strength, and ion aggregation may play a role in such systems since they can affect the rate of polymer chains interdiffusion over the surface of the crack. Despite the similar coordination geometry, a significant effect on maximum elastomer elongation before fracture can be related to differences in the M-L bond strength. Coordination to polymeric ligands to Zn^2+^, Fe^2+^ and Co^2+^ resulted in a maximum strain before fracture of 525%, 75% and 25%, respectively. The shorter coordination bond length, the stronger the association and the more brittle the network.

The pristine and healed samples of elastomers showed a similar trend in terms of maximum fracture strain and thermomechanical properties. Zn^2+^-crosslinked materials displayed a self-healing efficiency of 87% because the weak but dynamic coordination allows for fast regeneration of the coordination complex, while the polymers with more strongly coordinating Co^2+^ and Fe^2+^ self-healed with *η*_s_ of 61% and 71%, respectively. Strong interactions restrict the mobility of polymer chains, thus improving the mechanical strength but hindering self-healing ability. Rearrangement of ion aggregation may enhance the self-healing providing the ion aggregation is not intensive.

**Scheme 33:**
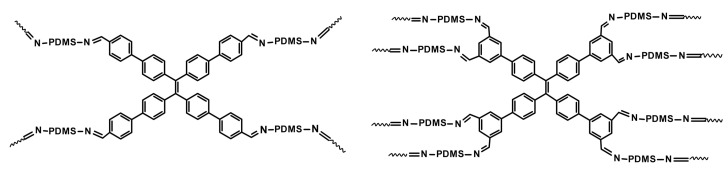
Structure of luminescent silicone elastomers.

The incorporation of aldehyde-modified tetraphenylene derivatives into PDMS networks via reaction with bis(aminopropyl) terminated polydimethylsiloxane (Mn 3000, 10,000, 16,000 g mol^−1^) resulted in luminescent elastomers of good thermal stability and mechanical properties, self-healable and recyclable due to the presence of dynamic imine bonds (Scheme 33) [81].

**Figure 17:**
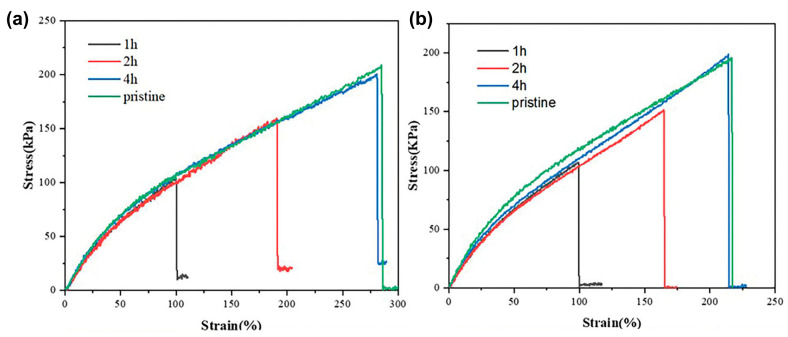
Tensile–stress curves of the pristine and pristine and healed elastomers (**a**) P1-3 and (**b**) P2-3. Adapted from [81], Copyright (2022), with permission from John Wiley & Sons, Inc.

The structure tetraphenylene derivatives and the molecular weight of the PDMS chains influenced the mechanical properties (Figure 17) and fluorescence emission (P1-3: *λ*_em_ = 530 nm and P2-3: *λ*_em_ = 525 nm at *λ*_ex_ = 365 nm). The damaged elastomers were effectively healed at room temperature and recycled under ambient conditions. The materials can be used in self-healable light-emitting devices.

A tetraphenylene derivative was used for the preparation of silicone elastomers dual crosslinked through combined dynamic covalent bonding through imine groups and metal (Zn^2+^, Cu^2+^, Fe^3+^) coordination bonds [38]. The primary single dynamic network was formed by coupling of bis(aminopropyl)-terminated PDMS and a tetraphenylene derivative modified with salicylaldehyde as concentrated crosslinking sites. The second crosslinking level was obtained by the incorporation of metal ions and their coordination to imine/phenol ligands. Furthermore, the branched aryl derivatives contributed the aggregation-induced fluorescence (AIE) emission effect due to the restricted intramolecular motion. The fluorescence response was specific to different metal ions, making the elastomeric network a valuable material for use in light-emitting devices.

The introduction of metal ions affected the properties of the silicone networks, changing their thermal, fluorescence and mechanical features. The effect could be adjusted by the type and content of metal ions involved because of the different coordination structures and metal bonding strengths. The stretchability and tensile strength of the dual crosslinked elastomers were enhanced compared to pristine silicones. Additionally, the reversibility of dynamic imine bonds and metal coordination sites provided self-healing properties and reprocessability.

The dual networks required more specific curing conditions than the single-crosslinked elastomer P-0 (99% efficiency of S-H after 24 h at room temperature). P-Zn-2 recovered only about 25% of the original tensile strength under the same conditions because a higher crosslinking density hampered the healing. S-H was improved at 70 °C due to diffusion enhancement resulting in faster reformation of imine bonds and metal–ligand complexes. The effectiveness of self-healing depended also on the type of metal ions (94.6%, 87.6% and 96.3% of the original tensile strength was restored, respectively, in elastomers P-Zn-2, P-Cu-2 and P-Fe-1.33). 

### 3.3. Self-Regeneration of Multifunctional Silicone Elastomers Involving Boroxine Chemistry

**Scheme 34:**
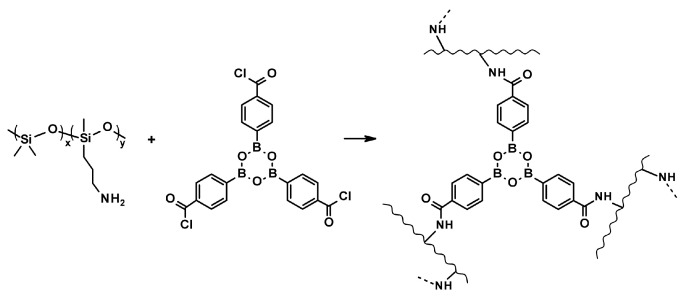
Dynamic crosslinking of siloxanes in PDMS-BN polymer network.

The boron–oxygen linkages bring in a unique combination of high bond dissociation energy and tuned response mechanism to self-healing systems. Boroxines are tripodal dehydration products of organoboronic acid. The boroxine/boronic acid equilibrium is readily shifted in the presence of a Lewis base (or water) or following a temperature change. Therefore, boroxines are often used as dynamic covalent crosslinks in high-strength room-temperature healable and recyclable high-strength materials. In addition, thermodynamically stable whilst kinetically labile nitrogen–ligation of boroxine ring structures increases the system dynamics at room temperature. These features make boroxines valuable dynamic crosslinks that have been incorporated into multifunctional S-H silicones for the synergistic effect of multiple dynamic bonds (Scheme 34) [82].

**Figure 18:**
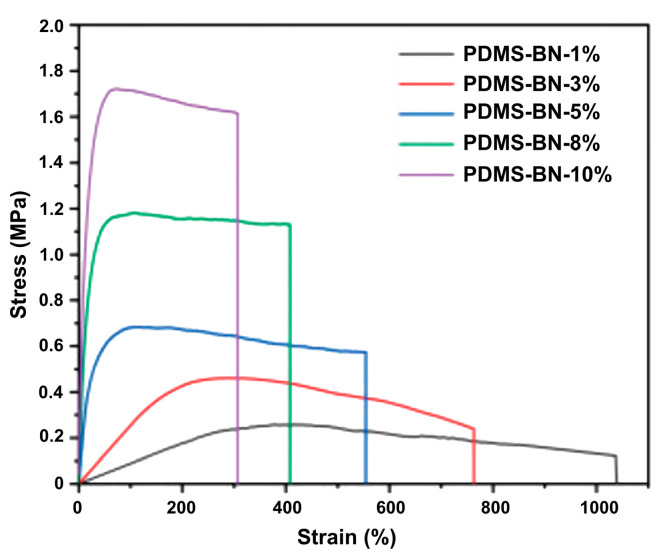
Stress–strain curves of PDMS-BN polymer network with different BN content. Adapted from [82], Copyright (2021), with permission from John Wiley & Sons, Inc.

The primary network formed by amidation provided good molecular mobility in PDMS-BN. The long-range intermolecular and intramolecular B—N coordination that involved the boroxine ring structure, increased the crosslinking density (secondary network). Hydrogen bonds additionally promoted the density of the dynamic network and acted as sacrifice linkages. When the PDMS-BN elastomer was subjected to external forces, dynamic breaking and reconstructing of hydrogen bonds and B—N coordination occurred. It allowed the stretched network to adapt to the applied forces. Energy dissipation due to the bond exchange was evidenced by hysteresis after multiple mechanical cycles. As a consequence, the overall mechanical properties were improved stress–strain curves. PDMS-BN with different contents of boroxine rings proved their influence on the network toughness (increasing with the increased amount of boroxine ligands) (Figure 18).

The elastomers exhibited good mechanical properties (*σ*_max_ up to 1.72 MPa, ε_max_ up to 307%, Young’s modulus up to ~11.2 MPa, and toughness up to ~4.9 MJ m^−3^ with 10% BN content). Intermolecular and intramolecular nitrogen-coordinated boroxines were involved in a synergetic dynamic mechanism leading to autonomic self-healing at room temperature with ~96% efficiency after 48 h. A sample with a lower content of BN (8%) healed more effectively than BN 10%, because of the lower amount of crosslinking points and easier chain diffusion. The transparent polymer was completely recyclable via crushing/molding or disassembling/casting and exhibited good adhesive properties and fluorescence–quenching response to Fe^3+^ ions. It can be used for Fe^3+^ sensors in special applications that require healability.

**Scheme 35:**
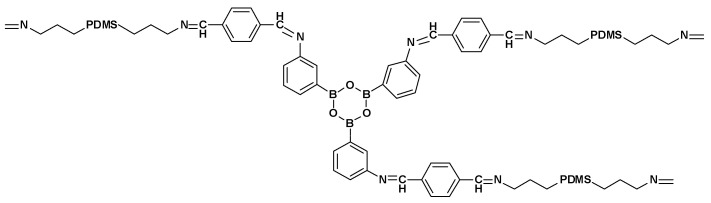
Chemical structure of siloxanes crosslinked with boroxine and imine bonds.

Boronic structures were also employed for the 3D crosslinking of α,ω-aminopropyl terminated polydimethylsiloxane (PDMS, Mn = 2000), through the Schiff base reaction and the dehydration reaction in a system containing terephthalaldehyd and 3-aminophenyl–oronic acid (APB) (Scheme 35) [83]. The self-healing dynamic network was based on boroxines, hydrogen bonds and imine bonds formed in the Schiff base reaction that linked both PDMS chains and phenylboronic structures to the aryl rings of TA derivatives.

Good mechanical performance of tensile strength *σ*_max_ of 2.54 MPa and ε_max_ of ~275% were achieved. The dissoluble and stretchable network self-repaired at room temperature with ~92% efficiency whereas 95% self-healing was noted at increased temperatures and ~85% in water. The polymer system was compounded with MWCNTs to prepare a hybrid composite for self-healing flexible electronic devices or sensors of changes in tensile and bending angles with the electric resistance.

**Scheme 36:**
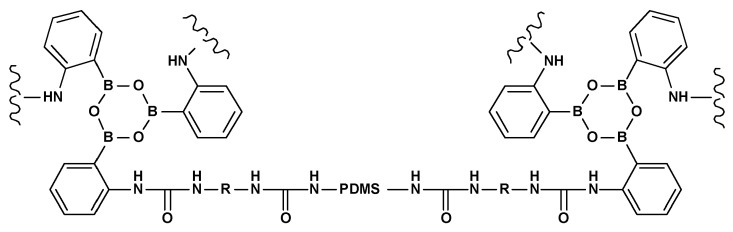
Chemical structure of APDMS-MDI-IPDI-Bs.

The boroxines were also used for supramolecular crosslinking of PDMS with urea segments (APDMS-MDI-IPDI-Bs) (Scheme 36) [84]. A synergistic effect of two types of hydrogen bonds with different strengths that involved urea groups (alkylaryl urea (MDI) and dialkylurea (IPDI)) and nitrogen-coordinated boroxine ring structures was observed along with microphase separation.

The hydrophobic elastomer displayed excellent weather and water resistance, *σ*_max_ up to ~3.35 MPa, ε_max_ up to 316%. Both mechanical properties and the S-H effect depended on the ratio of two types of urea motifs. The formation of hydrogen bonds between different urea groups depended on the steric hindrance. The importance of π–π stacking between the benzene rings in such systems should be stressed as it helps to achieve a tightly ordered arrangement of macromolecules that can improve the mechanical strength of the network. However, high loading with hydrogen-bonded MDI enhanced phase separation and crystallization in the siloxane matrix. IPDI regulated the hydrogen bond density in the silicone network. Nevertheless, in the absence of MDI the internal structure of IPDI-based networks was not stable enough to have good mechanical strength.

**Figure 19:**
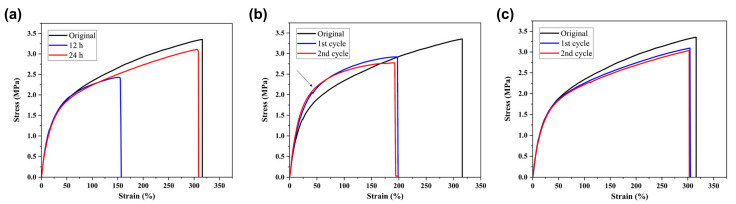
Stress–strain curves of APDMS-MDI_0.3_-IPDI_0.7_-B (**a**) after solvent (THF/H_2_O)-assisted healing, (**b**) formed by compression at room temperature, (**c**) disassembled and remolded with aqueous THF. Adapted from [84], Copyright (2023), with permission from John Wiley & Sons, Inc.

Almost 95% self-healing efficiency was noted after 24 h at room temperature when a small amount of THF/H_2_O (9:1 *v*/*v*) was added to the aligned incisions and after the drying process (Figure 19). The solvents were necessary to regain mobility of the hydrogen-bonded polymer segments. A new crosslinked structure was repeatedly reformed indicating high recyclability of the network. Interestingly, ethanol-assisted healing was less effective because of its incompatibility with the hard phase. On the contrary to the solvent-assisted self-healing, the effect of thermal treatment was rather poor. A sufficient simultaneous flowability of hard and soft phases in the material is required for S-H. It could be obtained at T > their *T*_g_, which is around 90 °C for the hard phase. At 90 °C, *η*_s_ improved, but was still less effective than the solvent-assisted healing.

## 4. Smart Applications of Self-Healing Polysiloxanes

In recent years, the research attention polysiloxanes attracted as the materials of choice for various emerging technologies was tremendous [4]. A rapid improvement in bulk modification strategies can be observed as well as the design of a new generation of PDMS-based smart materials, including flexible wearable electronics, sensors, coatings or e-skin [85]. The suitability of particular systems for various applications was discussed earlier in this review. In this chapter, we provide a concise summary of the information presented in the previous sections, that were mainly devoted to the chemistry and properties of specific groups of S-H silicones.

### 4.1. Polysiloxanes for Self-Healable Electronic Skin Applications

On-skin electronics should maintain the electrical function and simultaneously mimic the mechanical properties of biological systems. Materials designed for wearable artificial electronic skin applications (soft robotics and interface materials for on-skin electronics or human–machine interfaces) should thus display high mechanical flexibility, high breathability and a rapid mechanism of intrinsic autonomous self-healing that would provide efficient regeneration in unfavorable environments or after damage [86]. Preferably, such materials should be also capable of intelligently sensing environmental stimuli [87].

Siloxane polymers are suitable for electronic skin applications because of their biocompatibility, good air permeability, appropriate mechanical properties (modulus and stretchability) and chemical stability [2]. Several examples of hybrid S-H polysiloxanes designed or potentially suitable for the use as electronic skin have been already cited [61,65,67,77]. Another illustration can be PDMS combined with polyborosiloxanes (PBS) in interpenetrating “solid–liquid” elastomers (SLEs) that can mimic the velocity-sensitive ability of the human skin [88]. The dynamic network is formed by boroxine bonds whereas the polysiloxane chains form a permanent covalent scaffold. This system was elastic as well as structurally stable, stretchable and self-healing. The mechanical properties were dependent on strain rates. Superstretchability of up to 2100% and recovery of the initial shape upon unloading was noted. The dissociation and association of the dynamic network was found to be time-dependent and therefore the modulus of SLEs varied with strain rates. The introduction of carbon nanotubes (CNT, 5 wt.%) provided electric conductivity that was also responsive to strain rates. This system was used to fabricate strain–rate-responsive sensors (mechanoreceptors) with the ability to distinguish different contact velocities and mimicking the touching-rate sensibility of skin [88]. PDMS-dithiothreitol block polymers were shown to self-heal rapidly due to their high mobility and efficient formation of hydroxyl and boronate ester dynamic crosslinks [89]. The polymer not only presented a satisfactory mechanical strength (up to 0.43 MPa) and high stretchability (up to 1500%) but also recovered completely its original mechanical features at room temperature with an ultrafast rate in ambient environments (only ≤30 s after damage for 100% recovery). It also showed reconfigurability to any surface and had excellent self-adhesiveness (~10^2^ N m^−1^) to various surfaces both in air and under water. Moreover, the material was used for the fabrication of on-skin electrophysiological (EP) electrodes that displayed stable signal collection in air and under water, even during motion. Only 2 s were required to recover 100% conductivity without any external stimuli.

### 4.2. Flexible Strain Sensors

Self-healing, flexible pressure and strain sensors are also greatly promising for intelligent wearable electronic devices. High sensitivity, long-term durability and stretchability are required for such applications. Their self-healing effectiveness depends on the structure of a conductive network, mechanical properties of the matrix and effectiveness of the self-healing mechanism [90]. In addition to earlier cited examples [35,59,66,68,71,83], a stretchable hyperbranched polysiloxane elastomer with self-healing features based on D-A chemistry should be mentioned [91]. It demonstrated good tensile properties and robust mechanical strength (0.87 MPa) as well as high sensitivity to human motions. The self-healing efficiency achieved 85% in a relatively short time (thermal treatment at 130 °C for 10 min and 80 °C for 48 h) and admixing carbon black (CB) as the conductive filler by the solution blending–casting method enabled application of the composite in wearable flexible sensors. The content of CB affected the mechanical strength of the composite but also adversely the self-healing effectiveness. However, thermal healing may not be the best option for a wearable intelligent systems.

More efficient seem to be the sensors formed with the use of silicones crosslinked by dynamic bonds reversibly active at room temperature, especially multicomponent systems. A conductive bilayer strain sensor made of polysiloxane self-healing by acid–amine interactions and containing a thin film of carboxyl-functionalized carbon nanotubes was shown to be highly sensitive and self-adhesive [92]. The ultrasoft and self-adhesive polysiloxane substrate can provide a sufficient comfort in contact with the human skin. The sensor exhibited linearity (regulated by the thickness of the CNT layer), low hysteresis and long-term durability with a gauge factor of 33.99 at 55% strain. Human motions of various strength, including bending/unbending of fingers or knees but also coughing and swallowing, were detected with satisfactory sensing performance.

A dynamic microphase separation due to the combined action of hydrogen bonding, disulphide bonds and π–π interactions was exploited to furnish S-H polysiloxane elastomers with a tuneable tensile strength of 1.89–3.33 MPa, stretchability of 347–1722%, and an extreme fracture toughness 28.6 MJ/m^3^ [58]. A “sandwich-structure” flexible sensor device for detecting human motions was prepared with this material. Another example can be a strain sensor of high sensitivity, short response time and long-term durability and good mechanical integrity (672% elongation at break, 1.41 MPa tensile strength and Young’s modulus of 0.39 MPa) that was prepared using self-repairable crosslinked elastomer substrate and conductive layer of CNT [93]. The self-healing effect in this case was based on the dynamic disulfide, imine and hydrogen bonds (91% healing efficiency) whereas the mechanical parameters resembled those of human skin. The sensor demonstrated an effective electrical conductivity and sensitivity to various human body motions (gauge factor (GF) = 24.1), short response time (120 ms) and long-term durability (4000 cycles). 

### 4.3. Coatings

The term “smart coatings” refer to a class of materials used for the preparation of tailored functional coatings that can respond to certain intrinsic or extrinsic stimuli [94]. Smart and self-healing polymeric materials are of high interest for adjustable protection against the corrosion of metallic and non-metallic surfaces [95]. Smart ice-phobic coatings with responsive behaviors to external stimuli, such as changes of temperature, electrical and magnetic fields or pH are also of high interest [96]. The most recent trends in surface protection technologies include the formation of hydrophobic and superhydrophobic coatings or introduction of anti-fouling agents. Self-healing processes are required for the repair of coated areas damaged by ageing or action of aggressive events either by mending the defects or by inhibition of the corrosion process. In the earlier parts of this review, we have discussed the suitability of various S-H polysiloxane materials as smart protective coatings [46,62,63,74,75,78].

### 4.4. Self-Healing of Multifunctional Silicone Elastomers with Antimicrobial Properties

New trends in the design of antimicrobial silicone elastomers aim to develop new multifunctional materials with a range of useful properties including their recyclability and self-repair [7]. High-performance antimicrobial and antifouling materials with an in-built ability to recover mechanical strength can be used as fouling-release and corrosion-resistant polymer coatings. For example, flame retardant, self-healing and recyclable hybrid materials with >90% antimicrobial efficiency were obtained with PDMS, 2,2-bis(hydroxymethyl)propionic acid as the intermediate chain extender and crosslinked by interactions with tannic acid that is a natural antimicrobial substance [97]. The products exhibited good mechanical and tensile properties, with *σ*_max_ up to 7.5 MPa and ε_max_ up to 1540%. The mechanical properties and S-H mechanism was related to the dual action of H-bonding interactions and multiple dynamic phenol carbamate bonds with tannic acid macromolecules. The carbamates dissociated reversibly at 120 °C and a complete recovery was achieved on cooling (>85% after 18 h at 85 °C).

Another approach that can influence S-H ability, mechanical strength and antimicrobial properties is based on the application of nanoparticles of metal ions and oxides, especially zinc derivatives [98]. Although it can adversely affect chain diffusion and self-repair efficiency, dynamic thermally reversible ionic bonds can provide such polymers with recyclability and shape memory. For example, the tensile strength of PDMS-polythiourethane (PTU) composites was increased by ~20% while elongation at break was enhanced of ~30% on addition of 1 wt.% zinc oxide micro/nanoparticles. At this amount of t-ZnO, the phase structure containing microdomains was most homogeneous. Antimicrobial hydrophobic coatings of improved adhesion were also obtained with PDMS end grafted with 2-(2-benzimidazolyl)ethanethiol and zinc ions [99]. The synergistic SH effect was achieved by combination of dynamic Zn^2+^-imidazole coordination complexes and hydrogen bonding.

**Scheme 37:**
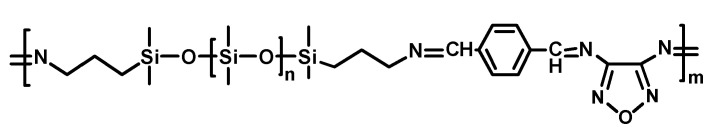
Chemical structure of PDMS-TA-DAF.

The antimicrobial and antifouling properties of S-H silicones are likewise very important for smart wearable electronics and robotics. ZnO nanoparticles grafted on the surface of MWCNTs were applied as microwave absorbers in a silicone-based composite self-healable via Diels–Alder chemistry [100]. The combined action of dynamic imine and hydrogen bonds with the coordination of zinc ions was reported for a silicone elastomer prepared with α,ω-aminopropyl terminated polydimethylsiloxane (PDMS, Mn = 3000), terephthalaldehyd and 3,4-diaminofurazan (DAF) (Scheme 37) [101].

**Figure 20:**
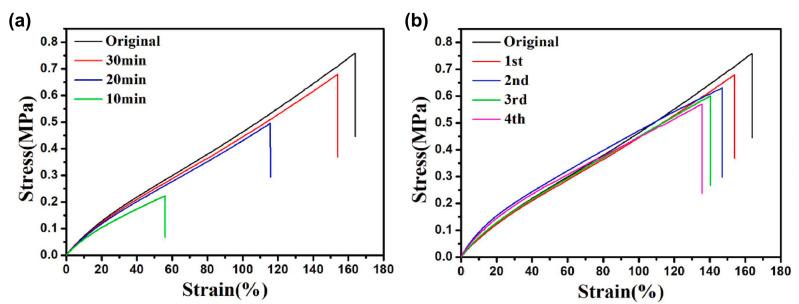
The stress–strain curves of PDMS-TA-DAF-Zn before and after S-H for different time (**a**) and after four cycle repair times (**b**). Adapted from [101], Copyright (2021), with permission from Elsevier.

The properties of this elastomer were further enhanced by the coordination of Zn^2+^ with nitrogen and oxygen atoms of 3,4-diaminofurazan of 1,2,5-oxadiazole structure. The elastomer was effectively repaired at 100 °C with the first *η*_s_ of 89.5% due to the synergistic action of imine bonds and zinc ion coordination bonds (Figure 20). The antibacterial efficiency of the material against *E. coli* and *S. aureus* was 99.9993% and 99.9997%, respectively. This significant effect is related both to the presence of biologically active 1,2,5-oxadiazole in the 3,4-diaminofurazan structure and the effect of zinc ions that helped to achieve the antibacterial ability.

## 5. Conclusions

In recent years, various chemical strategies have been investigated to enable the self-healing of silicone elastomers, especially soft functional materials for smart applications. Autonomously healing polysiloxane materials have been developed by incorporating moieties that can dynamically crosslink polymer chains, including those capable of participating in weak supramolecular interactions or containing specific covalent bonds. This approach turned out to be very successful in silicone elastomers due to their high segmental chain mobility that facilitate the reconnection of active species and regeneration of the damaged dynamic bonds. Under stress, dynamic interactions in siloxane elastomers are able to dissipate energy through reversible bond breaking. The synthesis of elastomers with well-controlled properties and the effect of interphase interactions between polymer chains are hot topics in the field of nanocomposite materials engineering.

Polysiloxane materials that can dynamically crosslink and self-heal through the action of molecules capable of generating weak supramolecular interactions or specific covalent bonds have been quite well studied and their advantages, as well as limitations, have been identified. Many of these systems require external stimuli, such as heat or light, catalysts, solvents or plasticizers. Although significant progress has been already made in this field, there is still room for further research and innovative concepts, especially new self-repairing materials for smart applications that require both rapid healing and excellent mechanical properties. 

In recent years, the development of new methods applied to the self-healing process of silicone elastomers has brought significant advances in the field of polymer composites and coatings. These new methods are based on the combination of several dynamic and supramolecular mechanisms to achieve a synergistic effect in improving self-healing and mechanical properties, as well as other specific characteristics (antibacterial properties, electrical conductivity) that can be introduced into such systems. In some cases, super-stretchability, adjustable Young’s modulus, elasticity and tensile strength, as well as fully recoverable mechanical performance, have been demonstrated. However, a deeper understanding of the various aspects of these materials’ performance is still needed.

This review presents the results of recent research dedicated to the design of multifunctional self-healing siloxane elastomers and the advantages of both non-covalent supramolecular interactions and dynamic covalent bonding in the engineering of next-generation silicone materials.

## Figures and Tables

**Table 1:** Composition and characteristics of the PDMS-Cat N polymers. Adapted with permission from [74]. Copyright (2019) American Chemical Society.

Sample	DOPA/IPDI/PDMS (mmol)	Mn	U Units	Cat Units	Modulus (MPa)	Hardness (MPa)
PDMS-Cat1-Zn	1/5.5/5	4450	6	2	172.0	10.7
PDMS-Cat2-Zn	0.5/5.25/5	8387	9	2	136.7	7.0
PDMS-Cat3-Zn	0.25/5.125/5	20,640	20	2	90.2	4.5
PDMS-Cat1-Ca	1/5.5/5	4450	6	2	162.5	12.4
PDMS-Cat1-Co	1/5.5/5	4450	6	2	166.9	13.1
PDMS-Cat1-Fe	1/5.5/5	4450	6	2	190.3	16.4

U—urea; Cat—catechol.

## Data Availability

Not applicable.
